# Definition and conceptualization of the patient-centered care pathway, a proposed integrative framework for consensus: a Concept analysis and systematic review

**DOI:** 10.1186/s12913-022-07960-0

**Published:** 2022-04-26

**Authors:** Jean-Baptiste Gartner, Kassim Said Abasse, Frédéric Bergeron, Paolo Landa, Célia Lemaire, André Côté

**Affiliations:** 1 Département de management, Faculté des sciences de l’administration, Université Laval, 2325 rue de la Terrasse, Québec, QC G1V 0A6 Canada; 2 Centre de recherche en gestion des services de santé, Université Laval, Québec, QC Canada; 3 Centre de recherche du CHU de Québec, Université Laval, Québec, QC Canada; 4 Centre de recherche du CISSS de Chaudière-Appalaches, Québec, QC Canada; 5 VITAM, Centre de recherche en santé durable, Université Laval, Québec, QC Canada; 6 Bibliothèque-Direction des services-conseils, Université Laval, Québec, QC Canada; 7 Département d’opérations et systèmes de décision, Université Laval, Québec, QC Canada; 8 Université de Strasbourg, EM Strasbourg-Business School, HuManiS, Strasbourg, France

**Keywords:** Care pathway, Patient journey, Care process, Patient-centered, Healthcare management, Sustainable transformation, Learning health system, Concept analysis, Systematic review

## Abstract

**Background:**

Confusion exists over the definition of the care pathway concept and existing conceptual frameworks contain various inadequacies which have led to implementation difficulties. In the current global context of rapidly changing health care systems, there is great need for a standardized definition and integrative framework that can guide implementation. This study aims to propose an accurate and up-to-date definition of care pathway and an integrative conceptual framework.

**Methods:**

An innovative hybrid method combining systematic review, concept analysis and bibliometric analysis was undertaken to summarize qualitative, quantitative, and mixed-method studies. Databases searched were PubMed, Embase and ABI/Inform. Methodological quality of included studies was then assessed.

**Results:**

Forty-four studies met the inclusion criteria. Using concept analysis, we developed a fine-grained understanding, an integrative conceptual framework, and an up-to-date definition of patient-centered care pathway by proposing 28 subcategories grouped into seven attributes. This conceptual framework considers both operational and social realities and supports the improvement and sustainable transformation of clinical, administrative, and organizational practices for the benefit of patients and caregivers, while considering professional experience, organizational constraints, and social dynamics. The proposed attributes of a fluid and effective pathway are (i) the centricity of patients and caregivers, (ii) the positioning of professional actors involved in the care pathway, (iii) the operation management through the care delivery process, (iv) the particularities of coordination structures, (v) the structural context of the system and organizations, (vi) the role of the information system and data management and (vii) the advent of the learning system. Antecedents are presented as key success factors of pathway implementation. By using the consequences and empirical referents, such as outcomes and evidence of care pathway interventions, we went beyond the single theoretical aim, proposing the application of the conceptual framework to healthcare management.

**Conclusions:**

This study has developed an up-to-date definition of patient-centered care pathway and an integrative conceptual framework. Our framework encompasses 28 subcategories grouped into seven attributes that should be considered in complex care pathway intervention. The formulation of these attributes, antecedents as success factors and consequences as potential outcomes, allows the operationalization of this model for any pathway in any context.

**Supplementary Information:**

The online version contains supplementary material available at 10.1186/s12913-022-07960-0.

## Background

While having a performant healthcare system is a crucial issue for every country, the health sector operates in silos that need to be challenged. Indeed, many authors have pointed to fragmented care processes as a cause of breakdowns in the continuity of healthcare services [1], unnecessary waiting times [2, 3], flaws in the flow of information between the different episodes [4] and the realization of exams that may be superfluous [5]. This fragmentation results in a sub-optimal use of material and financial resources and unsatisfactory team management [4]. Based on this observation, several repeated calls to improve the quality and performance of healthcare services have been made since 2001 by national and international institutions such as the Institute of Medicine of America (IOM) in 2001 [6] and 2013 [7], the National Academies of Sciences, Engineering, Medicine in 2018 [8] and the World Health Organization (WHO) in 2016 [9] and 2020 [10]. These calls have progressively shifted from an injunction to improve quality based on criteria to provide safe, effective, efficient, timely, equitable and patient-centered care [6], to the development of models for the organization of health care and services that meet the current challenges of effectiveness and efficiency in healthcare systems. The WHO urges member countries to base their quality improvement policies on the entire continuum of care, taking into account at least the criteria of effectiveness, safety, equity, efficiency, integrated care and timeliness [11]. These calls also emphasize the need to improve care pathways by focusing on outcomes that matter to the patient from a clinical, quality of life and health system experience perspective [12–15], rather than on the needs of the production units. This change of perspective leads to the study of the redesign of performance evaluation models by focusing on the needs and expectations of the patient [16, 17]. The problem is that there is confusion about the definition and characterization of a care and health service pathway. Indeed, Bergin et al. [2] identified 37 different definitions of the term care pathway based on a review of the literature. Definitions and characteristics vary across countries and include multiple phases ranging from prevention or screening to cure or palliative care. This confusion has led to wide variability in the outcomes of these interventions, resulting in underutilization of care pathway improvement programs [2]. Furthermore, such confusion leads to great variability in the analysis and modeling of care pathways. For example, in their scoping review, Khan et al. [18] showed the great variability that exists among studies of oncology care pathways in both the phases of care represented, and their characteristics. The lack of a common definition and clearly defined criteria leads to a lack of standardization, resulting in an inability to conduct reliable comparative studies of care pathway programs internationally [19].

The Oxford Concise Medical Dictionary 10th ed. [20] and the Oxford Dictionary of Nursing 8th ed. [21] define, in a concise way, care pathway as “a multidisciplinary plan for delivering health and social care to patients with a specific condition or set of symptoms. Such plans are often used for the management of common conditions and are intended to improve patient care by reducing unnecessary deviation from best practice”. The concept of a care pathway is one originally used in the field of Health Operations Management, whose definition was proposed by Vissers and Beech [22]. However, these definitions seem to be too imprecise and address neither the aim nor the social reality of implementing such pathways. The European Pathway Association (EPA) adopts the more precise definition from the 2007 thesis of Vanhaecht [23]. However this has not yet led to an international consensus, as confusion over the concepts remains high. Moreover, this definition does not clearly define the antecedents or factors favoring the success of such interventions, the means by which to implement them or the best practices through which to support them; nor does it sufficiently take into account the importance of the patient-centered care and patient-centered services approach. Similarly, the proposed implementation models largely neglected the social reality and the social dynamic of organizations [24], resulting in major implementation difficulties, as care pathways still being considered as complex interventions [25, 26].

However, care pathway programs have recently demonstrated encouraging results in terms of reduced variation in care, improved accessibility, quality, sustainability, and cost effectiveness of care [2]. The definition we aim to develop through this research is significant and timely, in that it has the potential to guide the ongoing development, implementation, monitoring and evaluation of care pathway programs within the rapidly changing service and system contexts that we are experiencing. For example, the following initial barriers to the systemic and holistic implementation of care pathways have recently been removed. Firstly, limited access to valid and reliable data from multiple organizations [27] has been offset by a massive investment in Electronic Medical Records [28]. Secondly, the main difficulties in highlighting the complexity of the referral trajectory [29], frequently resulting from the clinicians’ perspective, have been overcome by proposing new approaches such as data mining or qualitative methods, focusing on the real care trajectory and the qualitative part of the patients’ experience [16, 17, 30]. Therefore, the evolution of knowledge and information technology and the investment of health systems in data-sharing infrastructure, as well as a definition of the levers of patient engagement and the advent of patient-centered-care and patient-centered services, make it possible to define a powerful model for improving them by placing the patient’s needs and expectations at the center of the care pathway. It is therefore the right time to define a recognized definition and an integrative conceptual framework that meets the demand for sharing knowledge internationally regarding the development, implementation, and evaluation of care pathways.

The concept of patient-centered care is defined as “care provision that is consistent with the values, needs, and desires of patients and is achieved when clinicians involve patients in healthcare discussions and decisions” [31]. This approach is known to provide benefits by improving health outcomes, patient satisfaction, but also to reducing health costs [32].

A preliminary search for existing reviews was conducted in Cochrane Database, JBI Database of Systematic Reviews and Implementation Reports and PROSPERO. Care pathways have been the subject of few reviews, but these were limited to a single pathology such as cancer in general [33], blunt thoracic injury [34], cardiovascular disease [35], adolescent idiopathic scoliosis [36] or for particular pathway phases [37]. In the end, focusing on a single condition is not entirely consistent with a patient-centered approach to care insofar as patients often have comorbidities. The only review that did not focus on one specific pathology was made in 2006 [38] and was interested in the concept of clinical pathway. Authors reviewed literature published within 3 years using only one bibliographic database. Therefore, the aim of this article is to propose an accurate and up-to-date definition of care pathway and to develop an integrative conceptual framework for the patient-centered care pathway concept in a holistic operational approach of the concept.

## Methods

### Combining systematic review, concept analysis and bibliometric analysis

To achieve a fine-grained understanding of the concept, we have chosen a hybrid method combining the systematic review, the concept analysis and the bibliometric analysis methodologies. We followed the latest PRISMA (Preferred Reporting Items for Systematic reviews and Meta-Analyses) statement for conducting and reporting a systematic review [39]. However, the systematic review methodology presents some limitations on the qualitative analysis of literature, hence derives our interest to use Concept analysis. Concept analysis [40] aims specifically to clarify a specific concept including a semantic field linked to a specific theoretical framework. This approach is based on eight steps allowing to: (1) select the concept, (2) determine the aims or purposes of the analysis, (3) identify all uses of the concept, (4) determine the defining attributes, (5) identify a model case, (6) identify additional cases, (7) identify antecedents and consequences and (8) define empirical referents. However, this method does not provide a systematic and rigorous procedure for identifying and selecting relevant literature. Therefore, we decided to combine the strengths of both methods to overcome the limitations of each. In order to make our analysis more robust and to base our inferences, specifically in the comparative analysis of the related concepts, we performed a bibliometric analysis allowing us to link the attributes of each of the concepts to make a comparison.

### Information sources and search strategy

We developed a search strategy, in collaboration with a Health Sciences Librarian who specializes in systematic literature review in healthcare, to identify relevant peer-reviewed studies. An initial limited search of MEDLINE and CINAHL was conducted, followed by analysis of the text words containing title and abstract and index terms used to describe the article. This informed the development of a search strategy that was tailored toward each information source. The search strategy was applied to the following databases: PubMed, Embase and ABI/Inform. The complete search strategy is provided in Additional file 1.

### Eligibility criteria

This review considers studies that focus on quantitative and/or qualitative data, with no limitation in terms of methodology. Our search focused on peer-reviewed scientific articles. Therefore, books, doctoral or master’s theses were excluded due to time and resource limitations. In order to guide the selection, we chose the Population, Context, Concept (PCC) mnemonic criteria [41]. The population considers all types of patients managed by healthcare delivery systems. The context studied is composed of healthcare providers in any geographic area, including all providers of primary, secondary, tertiary, and quaternary care. For the concept, this review focuses on theoretical and empirical studies that contribute to the definition and conceptualization of the different related concepts of care processes at the organizational or system level, such as care pathway, clinical pathway, patient journey and care processes. Quantitative, qualitative and mixed method studies involving a single episode of care limited in time (a one-time treatment) or space (a single hospital service/department) were excluded to the extent that care pathway involves multiple points of interaction over time [13, 42] and multiple organizational structures or intra-organizational entities along the care continuum [43]. In addition, studies with no theoretical or conceptual input were excluded. Finally, there was no language or geographic restrictions applied to the search, and the study period was limited from 1995 to 2020.

These studies were imported into the Covidence® software (version 2020). The team developed screening questions and forms for levels 1 (abstract) and 2 (full text) screening based on the inclusion and exclusion criteria. Two reviewers independently screened the titles and abstracts. In case of disagreement, two senior reviewers decided after analysis and discussion. Review author pairs then screened the full-text articles against inclusion and exclusion criteria. In case of disagreement, the same process as for the title and abstract selection was implemented. Reasons for excluding studies were recorded.

### Assessment of methodological quality

Because of the heterogeneity of the methods used in the selected articles, we decided to use a separate appraisal tool for each study type. The following appraisal tools were selected for their clarity, relevance, and because their items covered the most common assessment criteria comparing to other tools:

- For qualitative studies: the JBI Qualitative Assessment Research Instrument (QARI) [41]
- For surveys: the Center for Evidence Based Management (CEBMa) Appraisal Questions for a Survey [44]
- For descriptive cross-sectional studies: the Institute for Public Health Sciences 11 questions to help you make sense of descriptive/cross-sectional studies [45]
- For mixed-method: the scoring system for appraising mixed methods research [46]

No articles were excluded from this systematic review due to the weaknesses of their methodological quality, so as not to exclude valuable information [47].

### Data extraction and analysis

Descriptive numerical summary analysis followed the systematic review guidelines, and the following items were systematically extracted: Reference, Title, First Author country, Case country, Year of publication, Type of publication, Target patient population, Phases of the pathway included, People involved in the modeling process, Study parameters and level of analysis.

Qualitative data were extracted using MaxQDA® software (version 2020) by two independent analysts. The data extraction followed the concept analysis guideline [40] and the following items were systematically extracted: Variant concept studied, Concept uses, Concept definition, Concept attributes, Antecedents, Consequences and empirical referents. In order to develop a detailed analysis and arrive at a robust theoretical framework, we relied on general inductive analysis [48], consisting of coding, categorization, linking, integration and modeling. Each step has been validated by at least two senior authors.

A bibliometric analysis was performed with the complete texts of the 44 selected studies using Vosviewer® software (version 2020).

The systematic review was reported following the latest PRISMA statement for conducting and reporting a systematic review [39] and mobilized the PRISMA 2020 checklist (see Additional file 2).

## Results

**Fig. 1:**
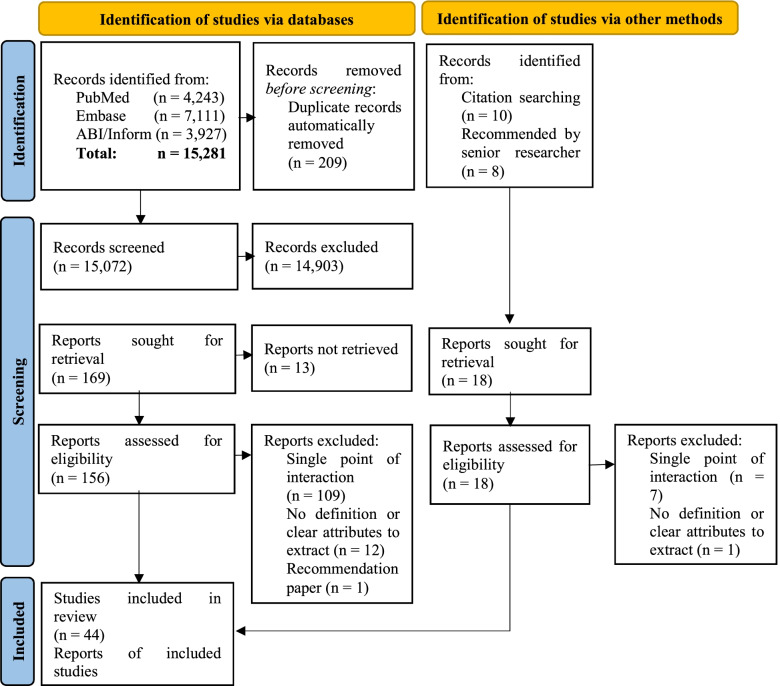
PRISMA 2020 flow diagram of the systematic review process

The interrogation of the three databases resulted in 15,281 articles. Figure 1 details the selection process following the PRISMA 2020 statement [39]. After deleting the duplicates, 15,072 records were reviewed but only 44 publications ultimately met the inclusion and exclusion criteria.

### Description and methodological quality appraisal of studies

A summary table containing a brief description of selected studies and their evaluation results for methodological quality is presented in Table 1. Quality appraisal of selected studies is presented in Additional file 3.

**Table 1:** Summary of the characteristics of included publications

Author(s), year	First author country	Study design	Terminology used	Target patient population	Level of the concept	Setting	Participant demographics	Type of data	Methodological quality appraisal
Aspland et al., 2020 [49]	United Kingdom	Literature review	Clinical pathway	NA	Organizational	NA	NA	Number studies (*n* = 175)	NA
Bergin et al., 2020 [2]	Australia	Literature review	Optimal care pathway	Cancer care	Systemic	NA	NA	NR	NA
Busari et al., 2020 [50]	The Netherlands	Qualitative	Care pathway	Decubitus ulcer	Organizational	St. Elisabeth Hospital, Curaçao	Medical specialist Nurse Paramedical professional Management & educational staff	Participants (*n* = 33) Interviews (*n* = 10)	Medium
Carayon et al., 2020 [24]	United States of America	Perspective article	Patient journey	NA	Systemic	NA	NA	NA	NA
Cherif et al., 2020 [42]	France	Qualitative	Patient journey	Breast cancer	Systemic	National forum for breast cancer patients	Breast cancer patients	967 reviews were collected over a year	Medium
Devi et al., 2020 [51]	India	Literature review	Patient journey	Non-communicable Diseases	Systemic	NA	NA	NR	NA
Elkhuizen et al., 2020 [52]	The Netherlands	Survey	Patient journey	Diabetes type 2	Systemic	diabetes type 2 in a region of The Netherlands	910 patients	quality of life (EQ5D), service satisfaction, experiences	Medium
Hutchinson et al., 2020 [3]	Australia	Mixed-method	Care pathway	Refractory epilepsy	Systemic	New South Wales, Australia	Medical specialist, patients	Interviews (*n* = 22), observations (n = 10), surveys (*n* = 20)	Medium
Kempa-Liehr et al., 2020 [43]	New Zealand	Qualitative	Care pathway	Appendicectomy	Organizational	North Shore Hospital, New Zealand	NR	NR	Low
Ocloo et al., 2020 [53]	United Kingdom	Qualitative	Patient-centred care	Stroke and hip fracture	Systemic	King’s Fund (11 hospitals)	Doctors, nurses, senior managers, service improvement specialists and patient representatives	Participatory action research, documentary analysis, participatory steering groups (*n* = 7), focus group (*n* = 8) and interviews (*n* = 47)	High
Seguin et al., 2020 [35]	United Kingdom	Literature review	Care pathway	Cardiovascular diseases	Systemic	NA	NA	Number studies (*n* = 15)	NA
Alkandari et al., 2019 [5]	United Kingdom	Qualitative	Patient journey	Peripheral Neuropathy	Systemic	Ibn Sina Neurology and Neurosurgery Hospital	Patients	Interviews (*n* = 25)	Medium
Ayachi et al., 2019 [54]	Tunisia	Survey	Care process	NR	Organizational	UHC Habib Thameur and Charles Nicolle Hospital	Hospital staff, head of department, engineers and patients.	NR	Low
De Belvis et al., 2019 [55]	Italy	Descriptive cross-sectional stud	Clinical pathway	Ischemic stroke	Systemic	Italian teaching hospital	483 stroke patients	Electronic records (*n* = 483)	High
Gualandi et al., 2019 [14]	Italy	Qualitative	Patient journey	Hip and knee replacement surgery	Organizational	250-bed Italian teaching hospital	Patients and professionals	Interviews (n = 20), patient shadowing (n = 8)	High
Louis et al., 2019 [56]	United States of America	Qualitative	Patient-centered care	Breast cancer	Systemic	Three health systems	Physician, inpatient and outpatient nursing, patient navigation, clinical trials, genetics, and care coordination.	Interviews (*n* = 30)	High
Meyer, 2019 [57]	United States of America	Mixed-method	Patient journey	Multiple chronic conditions	Systemic	Large health system and its care partners	NR	NR	Low
Schildmeijer et al., 2019 [15]	Sweden	Qualitative	Standardized care pathway	Prostate Cancer	Systemic	Midsized hospital in southeast Sweden	Professionals and patients	Interviews (*n* = 14)	Medium
Fung-Kee-Fung et al., 2018 [4]	Canada	Mixed-method	Care process	Lung Cancer Care	Systemic	A regional Community of Practice, Ottawa	Professionals, patients and caregivers.	Interviews (*n* = 68), Quantitative data (NR)	Low
Kelly et al., 2018 [1]	Australia	Qualitative	Patient journey	Aboriginal patient	Systemic	Adelaide region	Patients and their families, healthcare professionals, managers and support workers	Participatory action research from 2008 to 2015, interviews (*n* = 21) and focus groups (n = 17).	Medium
Mohr et al., 2018 [58]	Czech Republic	Qualitative	Patient journey	Schizophrenia	Systemic	Board of the European Psychiatric Association	Representatives of patient and family organizations, health policy and economic experts, and drug companies	Interviews (n = NR) and focus group (n = NR)	Medium
Ponsignon et al., 2018 [13]	France	Qualitative	Patient journey	Cancer	Systemic	UK Patient Opinion data	Patients	Stories (*n* = 200)	Medium
Aziz et al., 2017 [59]	Malaysia	Qualitative	Integrated care pathway	Stroke	Systemic	Ministry of Health, Malaysia	Physicians, nurses and managers.	Focus group (n = 2)	Medium
Combi et al., 2016 [60]	Italy	Qualitative	Care pathway	Chronic Obstructive Pulmonary Disease	Systemic	Region of Veneto	General practitioner	Focus group (n = NR), expert Interviews (n = NR), users’ input.	Low
Gillespie et al., 2016 [61]	United Kingdom	Descriptive cross-sectional stud	Patient journey	Stroke	Systemic	Belfast City Hospital	Patient Administration System data	5-year retrospective dataset (*n* = 1995)	High
McCarthy et al., 2016 [30]	Ireland	Qualitative	Patient journey	Hypertension during Pregnancy	Systemic	NR	Multidisciplinary practitioners	Focus group (n = 4)	Medium
Shaw et al., 2016 [62]	Australia	Survey studies	Clinical pathway	Anxiety and depression in adult cancer	Systemic	Australian oncology and psycho-oncology	Physicians	Dataset (*n* = 247)	Medium
Valentijn et al., 2016 [12]	The Netherlands	Literature review	Value-based care	Chronic kidney disease	Systemic	NA	NA	Number studies (*n* = 26)	NA
Walker et al., 2016 [63]	New Zealand	Descriptive cross-sectional stud	Patient journey	Breast Cancer	Systemic	North Shore hospital	Wide range of stakeholders	Dataset (*n* = 72)	Medium
Beausejour et al., 2015 [36]	Canada	Descriptive cross-sectional stud	Care pathway	Suspected adolescent idiopathic scoliosis	Systemic	Paediatric orthopaedic clinics of south-western Quebec	Children and accompanying parents	Between February 2006 and August 2007, (*n* = 831)	High
Grenness et al., 2014 [64]	Australia	Qualitative	Patient-centred care	Hearing aids	Systemic	Victoria	Adults who had owned hearing aids for at least 1 year	Interviews (n = 10)	Medium
Van Citters et al., 2014 [65]	United States of America	Mixed-method	Clinical pathway	Total joint arthroplasties of the hip and knee	Systemic	County in southeast Sweden	Clinical, academic, and patient stakeholders	Interviews (*n* = 64), hospital databases (n = 4).	Medium
Evans et al., 2013 [66]	Canada	Qualitative	Disease Pathway Management	Lung Cancer	Systemic	Cancer Care Ontario	Primary care, public health, occupational medicine, oncology, and supportive services, and patients and caregivers.	Focus group (n = 25)	Medium
Huang et al., 2012 [67]	China	Qualitative	Patient-centered care	Anemia in pregnancy	Organizational	NR	Professionals	NR	Low
Tehrani et al., 2012 [68]	United Kingdom	Qualitative	Clinical pathway	Gynecology	Organizational	NR	NR	NR	Low
Vandborg et al., 2012 [69]	Denmark	Qualitative	Care process	Gynecological cancer	Systemic	Department of Gynecology and Obstetrics, Odense	Patient, general practitioner, and hospitals professionals	Number of cases (n = 6)	Medium
Yang et al., 2012 [70]	China	Perspective article	Clinical pathway	NR	Organizational	NA	NA	NA	NA
Manchaiah et al., 2011 [71]	United Kingdom	Qualitative	Patient journey	Hearing impairment	Systemic	Swansea Hard of Hearing Club	Patients (*n* = 32)	Focus group (n = NR)	Medium
Yamazaki et al., 2011 [72]	Japan	Qualitative	Clinical pathways	NR	Organizational	Saiseikai Kumamoto Hospital and Fukui General Hospital	NR	Documents analysis, participant observation, and interviews	Low
Vanhaecht et al., 2010 [25]	Belgium	Literature review	Care pathways	NA	Organizational	NA	NA	Number studies (n = NR)	NA
Allen et al., 2009 [73]	United Kingdom	Literature review	Integrated care pathway	NA	Systemic	NA	NA	Number studies (*n* = 9)	NA
Joosten et al., 2008 [74]	The Netherlands	Qualitative	Integrated care pathway	Mental Health Care	Organizational	Institute of Mental Health Care, Eindhoven	NR	NR	Low
De Bleser et al., 2006 [38]	Belgium	Literature review	Clinical Pathway	NA	Organizational	NA	NA	Number studies (*n* = 37)	NA
Bond et al., 2001 [75]	United Kingdom	Qualitative	Care pathway	Hip surgery	Organizational	Six orthopaedic departments in English hospitals	Managers, medical staff, clinical nurses and other professionals.	Interviews (n = NR)	Low

*NA* Criterion not applicable, *NR* Criterion not Reported

**Fig. 2:**
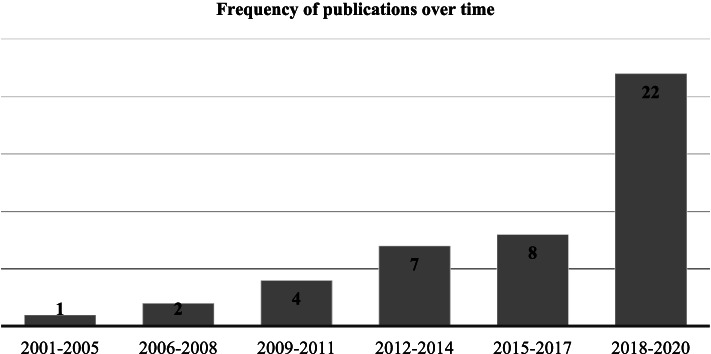
Frequency of selected publications over time

Published articles, describing care pathways as multiple points, in time and space, of patient interaction appeared in the early 2000s. However, most of this work has been published since 2010, with a progressive and growing interest, whatever the theoretical position, to reach 22 articles in the last 3 years (see Fig. 2).

The countries of the first authors interested in this concept are predominantly anglophone such as the United Kingdom (k = 9), Australia (k = 5), the United States (k = 4), and Canada (k = 3). Researchers from other countries are less represented.

Three types of publications were found; 34 were original research studies, eight were literature reviews and two were perspective studies. In the original research studies, 23 used a qualitative approach to study either the implementation of a care pathway program or patient experience of a care pathway, four used a descriptive cross-sectional approach, four used a mix-method approach and three used a survey.

Since the definition of the concept is still unclear and terminology is important, the studies meeting the selection criteria reported several terminologies. The most frequently used terms in the selected studies were the patient journey (k = 14) and the care pathway (k = 13) with their some country-specific modifications namely integrated care pathway mainly in the United Kingdom [73, 74], optimal care pathway in Australia [2] and standardized care pathway in Sweden [15]. The other terms used were clinical pathway (k = 8), patient-centered care (k = 4), care process (k = 3), disease pathway management (k = 1) and value-based integrated care (k = 1).

Studies focused mainly on the care of chronic conditions (k = 24), followed by acute diseases (k = 11). Of those with a chronic care focus, cancer was by far the most studied disease (k = 10), followed by stroke, hearing impairment and mental disease. Acute care studies covered, articular pathologies of the hip and knee, and pregnancy.

Concerning the level of the study, most addressed the systemic (k = 31) rather than the organizational (k = 13) level. Most authors, in their approach to the concept, largely focused on the treatment phase (k = 39), but some included, more or less, pretreatment and subsequent phases. Only seven articles took a global approach starting from the prevention phase and screening to survivorship or palliative care phase.

### Concept analysis results

The conceptual analysis followed an automatic data extraction method in the proposed main categories and then, after several iterations, resulted in a coding of subcategories grouped into main themes. The detailed results of the coding are presented in Additional file 4.

#### Concept uses

Uses of the concepts of care pathway have evolved in the literature over time with a strong tendency to focus on the care pathway at the systemic level. Main objectives have been improving quality and safety (k = 26), improving efficiency in the delivery of care (k = 24), optimizing the delivery process through an operation management point of view (k = 22) and integrating best practices through guidelines and evidence-based medicine (k = 17). These objectives were widely shared and present throughout the period. However, interest emerged in 2009 and quickly grew, in improving the patient experience through the analysis of the patient journey (k = 17). To a lesser extent, the goals of developing patient-centered care (k = 13), improving patient outcomes (k = 13), improving coordination of service delivery (k = 13), and standardizing care delivery (k = 12) were also present. Beyond standardization, reduced variation in care practices (k = 9) was not well addressed, nor was continuous performance assessment (k = 8). The aim of meeting the patient’s needs (k = 6) has been addressed more frequently in recent years, since its first appearance in 2011 [71], and is considered of crucial importance by some authors. Other concept uses were proposed, such as to improve interprofessional collaboration (k = 5), support changes (k = 5), support clinical decision making (k = 4), improve communication (k = 3), consider needs of healthcare workers, improve referral system, define shared purposes and meaningful objectives (k = 2), monitor staff compliance, support the knowledge management, improve patient and family member access to information, adopt a system approach and understanding power dynamics and relational factors (k = 1). As described previously, these concept uses came mainly from the chronic disease care context, although acute care was also represented.

#### Defining attributes

Definitional attributes are features commonly encountered in definitions of the concept or frequently used to describe it [40]. Twenty-eight attributes were inductively extracted and categorized into seven main themes, ordered by level of empirical importance: (1) The centricity of patients and caregivers; (2) the positioning of professional actors involved in the care pathway; (3) the operation management through the care delivery process; (4) the particularities of coordination structures; (5) the structural context of the system and organizations; (6) the special role of the information system and data management; and (7) the advent of the learning system (k = 3).

#### Attribute theme 1: The centricity of patients and caregivers

Firstly, there has been a growing interest in the patient experience (k = 15), mainly through the concept of the patient journey [5, 13–15, 24, 30, 42, 51, 52, 58], which has progressively emerged as the third pillar of quality in healthcare with clinical effectiveness and patient quality and safety [30]. It is formed by all the interactions at the meeting point, or point of contact, between health services and patient [14, 30, 42, 51]. However, taking the patient experience into account is complex insofar as it requires a detailed understanding of what influences it. Therefore, some authors have defined the dimensions that can influence the patient experience as the temporal dimension, meaning that accessibility and short waiting times are valued [13, 15, 30, 42, 51], the spatial dimension [30], and the geographical position of the services [42], the emotional dimension [13, 30, 42] and the social and cognitive dimensions [13, 42]. All these dimensions can be the source of both positive outcomes [13, 30] and negative outcomes [15] or for socio-political authors, a feeling of considerable disempowerment [53]. Although authors are increasingly interested in it, the patient experience is still sometimes overlooked [14].

Patient information and education (k = 15) were addressed in numerous studies. Patient information contributes to the quality of the patient experience [3, 15, 36, 42, 53, 64, 71, 75]. Beyond the simple satisfaction, the provision of information, at an appropriate health literacy level, increases patient awareness [36, 51] and thus increases patient education. This results in a better detection of the symptoms at an early stage by the patient [3, 36], the development of the “expert patient” [51, 57, 58, 71], which aids adherence to treatment, supports shared decision-making [57] and improves self-management [51, 58]. However, many empirical studies showed there to be a lack of patient information throughout patient journeys [5, 14, 15, 42, 51, 53, 64].

Patient engagement (k = 15) was an important attribute of this theme in the more recent literature. The management by the patient of his or her care treatment plan has become increasingly important [24, 50, 51, 53, 67]. This translates into shared decision-making on care and treatment [3, 14, 24, 35, 51, 53, 55–58, 64, 65]. According to Devi et al. [51], this process can only be viable if supported by good information about treatment possibilities and possible outcomes. However, socio-political authors see this as a major issue of patient empowerment, which is “seen as a solution to many of the most pressing problems facing modern healthcare” [53].

Proposed only since 2014, and strongly present in the last 3 years, relationship as the basic need (k = 9) is also a subject of interest. Part of the patient experience, the relational quality reflects how patients perceive their interactions [13, 42]. Some empirical studies have shown that a poor relationship can negatively affect other processes and tasks [3, 5]. Therefore, quality of the relationship seems a fundamental prerequisite [14, 64]. For this reason, some authors have placed the notion of trust as essential to the quality of interactions and to the patient’s follow-up through the care pathway [3, 12, 58].

Patient and Public Involvement (k = 9) is part of these new topics. Its importance in the design and improvement of the care pathway is supported by some international organizations [9]. The objective is to improve the quality of care provided by assessing patients’ perceptions [12, 13]. In this way, the design of care delivery can be based on the real needs and expectations of patients [12, 13, 51, 56, 62]. However, some models have been criticized as tokenistic rather than being viable solution for balancing power between patients and health care providers [53].

Although the stated goal of care pathways incorporates an approach aimed at standardizing care practices, several authors have raised the need for individualized care (k = 8). Joosten et al. [74] saw a potential conflict between standardization and the demand for a personalized approach to healthcare. However, several authors have subsequently agreed that there is still room for individualization of care beyond the standardization [55], in particular through the definition of personalized treatment goals [51], or even maintaining flexibility in the interaction to better adapt to the patient’s specific needs [64, 65].

Developed only since 2016, the importance of psychosocial support (k = 8) has increased rapidly. Although the need has been clearly identified and documented [5, 15, 42, 58] and many international guidelines have integrated it, it seems that its translation within the care pathway is still complex [62] and no obvious answer was provided.

The inclusion of family and caregiver (k = 8) is also a new topic of the last 5 years which highlights the potential of family or caregivers involvement in decision-making [50, 51, 57, 65]; notably by supporting both the integration of information and personal decision-making [14, 15].

#### Attribute theme 2: The positioning of professional actors involved in the care pathway

Firstly, most authors consider the care pathway as a tool to develop patient-centered care (k = 18). The patient-centered care approach has a disease-specific orientation [25] and considers the patient as a real partner [51, 25]. In doing so, this approach recognizes an individual’s specific health needs and preferences as the driving force in all healthcare decisions [13, 51, 65, 67]. Thus, professional actors emphasize their accessibility and their attitudes and behaviors towards patients [13]. In addition, this approach considers the importance of integrating family and caregivers and is recognized as a necessary attribute of healthcare quality [65]. Finally, its implementation seems to improve patient satisfaction by moving toward an individualized therapy approach and personalized treatment goals [51].

Not surprisingly, multidisciplinary team-working (k = 17), and attribute which is consistent with previous definitions, is supported by several authors. The enrollment of all professional categories involved directly or indirectly in the care pathway at all steps is valued [2, 50, 75]. The multidisciplinary teamwork allows tackling the complexity of patient care across the pathway and developing a shared understanding supported by knowledge sharing among professionals [53, 72]. In addition, it allows outlining the optimal sequence and timing of interventions [38, 59] and to focus only on patient needs and engagement rather than on problems of a particular profession [56]. From an operational view, multidisciplinary care teams make it possible to share formal screening between disciplines [62]. Recently, multidisciplinary engagement was identified as a mandatory prerequisite for successful care pathway programs [24, 50].

Staff skills (k = 10) could be considered equally important for care pathways. However, they were not addressed in this literature before 2014. Authors gave little attention to technical skills, except to point out possible deficiencies, particularly in diagnosis [3, 13], but also in training [3]. Rather, authors focused almost exclusively on interpersonal skills [3, 12, 13, 15, 51, 64], which were considered critical, both in the relations between professionals [12, 15, 51, 56, 64] as well as those with patients and their caregivers [15, 51, 64]. Interpersonal skills could be seen as facilitators or barriers to the patient experience [64]. Some authors have recently suggested that peer cooperation was critical [5, 50, 56] and that creating a culture of mutual respect among both medical and administrative colleagues can ultimately improve the fluidity of care [3, 5].

Few authors have highlighted that the implementation of a care pathway leads professionals to examine their roles and responsibilities (k = 6). The need to define each step in the care process requires professionals to describe precisely the tasks and roles of professional actors [25]. In doing so, it creates a rare opportunity to step back from daily tasks and reassess competences, roles and responsibilities [12, 51, 73].

Finally, very recently, authors have been interested in the experience of staff (k = 2) in care pathway programs. These authors have demonstrated the link between staff experiences and their individual performance [24, 53]. They therefore support the idea that staff well-being is directly related to engagement and performance and, thus, a negative staff experience can influence patient, clinician, and organizational outcomes.

#### Attribute theme 3: The operation management through the care delivery process

This analysis has shown, unsurprisingly, that the process approach to care delivery (k = 23) was the core of the care pathway approach across the literature to date. From an engineering perspective, as define by the International Organization for Standardization, a process is “a set of interrelated or interacting activities that transforms inputs into outputs” (ISO 9000:2000 clause 3.4.1). Through this approach, the care process can be defined as an arrangement of tasks or actions sequenced in time resulting in a time matrix [24, 30, 38, 52, 60, 68, 25, 73]. What distinguishes the different process approaches to care delivery are the tasks and actions included with them. Some authors tend to focus on operational planning by treating tasks, actions and their timing through business processes [43, 49, 54, 60, 69], while other authors consider both the context of action through the physical and organizational environment [24, 30] and social dynamic through the experience of actors [24, 52, 53]. Through this approach to care processes, some authors focus on patients and caregivers [52] and other authors focus on human actors, both patients and caregivers and the professional actors involved in the care pathway [24]. In 2018, Ponsignon et al. [13] proposed to differentiate the direct, indirect and independent interactions (those disconnected from the delivery system), in care processes. Direct interactions constitute the points of contact between patients and the system, and so are responsible, along with indirect interactions, for the patient version of the pathway that some authors call the patient journey [5, 13, 30, 51, 53]. More recently, the complexity of the care process has led some authors to consider that the care pathway should involve pathway rules which control the process [70]. Thus, decision-making becomes a central element in the smooth running of the care pathway [60]. In addition, many authors consider that healthcare decisions and care pathways are intertwined so that it becomes imperative to co-design both care pathways and the decision-making activities [60].

The issue of process management for the delivery of care naturally raises the question of process modeling methods (k = 18). In the empirical articles, the use of the Business Process Modeling Notation (BPMN) developed by the Object Management Group seems to be progressively imposed, sometimes improved by decision modeling [4, 43, 54, 60, 68, 69]. The use of process mapping or flowcharts with sometimes less formal rules seems to be favored for global approaches to processes, especially for the patient journey, although some authors such as Combi et al. [60], have demonstrated that BPMN modeling was quite compatible with the systemic approach.

For healthcare service designers, the methods for building care pathways are important considerations. Several methods exist, but all involve the discovery of a different path, thus change is inevitable and change management a necessity. The initial method came mainly from the expertise of professionals through interviews, focus groups or Delphi methods [49, 59]. The advantage of collaboration with staff and experts is that more information can be gathered about certain decisions and possible variances from the pathway [49]. However, this method did not consider the real trajectory or the ideal pathway but rather the one integrating the constraints of the professionals. Since these early efforts, data driven approaches has developed considerably [43, 49]. Their advantage is that they inform pathway development from data derived factually and objectively from actual occurrences of the pathway [49]. Moreover, data on the perspectives of patients through experience mapping, interviews, focus groups or observations [5, 13, 30], and patient shadowing [53] can be integrated to better reflect the real trajectory and to define the ideal pathway according to the needs and expectations of patients and caregivers. However, this approach does not allow for the integration of context and organizational constraints. Finally, few authors adopt an approach that consists of comparing the experience of professionals and patients, making it possible to define the lived experience, the patient’s journey, and its confrontation with operational realities and constraints through the experience of professionals [1, 3, 4, 15, 65, 71].

Regarding the process of care delivery, the management of operations aims to integrate the organization of the delivery process with its ongoing improvement (k = 11) by focusing as much on analyzing the variations as on eliminating the wastes [74]. Process improvement tools serve as much to redesign the processes as define a workflow management system to monitor the care pathway [4]. The information generated [60, 61, 63] can be used for process re-engineering, objective reassessment or supporting non-clinical decision-making [60], such as the identification of bottlenecks [61, 67] or highlighting interfacing problems between organizations [61]. The output generated by the analysis of the process-related data allows defining standardized expedited diagnostic processes [4, 60]. Finally, the data obtained allows the use of simulation and optimization models. On this subject, Aspland et al.’s literature review [49] provides an exhaustive review of available methods.

#### Attribute theme 4: The particularities of coordination structures

In line with most of the definitions, the integration of the clinical practice guidelines, based on evidenced-based medicine, into the care pathway (k = 24) has been accepted since the beginning of such programs. The clinical decisions directly affect the flow of the care delivery process and thus the process performance and the quality of outcomes [60]. Therefore, the adherence to clinical practice guidelines must support decision-making [70, 73] and aid diagnosis and treatment in order to improve patient outcomes [50, 51, 58]. In 2010, Vanhaecht et al. [25] expressed concern about a lack of evidence-based key interventions within care pathways. The care pathway can be an effective method to integrate and guarantee the appropriate use of evidence-based interventions and clinical practice guidelines [55] and may help to overcome two limitations of clinical practice guideline use, which are emerging as key issues [60, 66]. Firstly, that they should not be followed blindly as they represent only explicit medical knowledge [67], but rather require integration of the contextual knowledge of healthcare professionals for appropriate use [72]. Secondly, it has been shown that physicians can be unaware of updates and changes to clinical guidelines [3], and so, integrating them into care pathway maps may improve guideline use and adherence. Finally, collectively integrating and discussing clinical practice guidelines appears to improve interprofessional collaboration and clarify roles [36], but also could benefit the involvement of patients in the co-design of the care pathway [35].

Some authors consider information continuity (k = 13) as a key factor. Not only because sharing information must support decision-making [60, 75] and facilitate communication [2, 12, 38], but more broadly because the disruption of the information flow can lead to coordination problems and easily avoidable costs linked to the repetition of examinations [5, 56, 59]. Therefore, the continuity of information must be supported to ensure sustainable health improvements [51, 70]. Some authors insist on the importance of defining an information medium throughout the pathway which is as accessible to care professionals as it is to patients and caregivers [65].

Recently, some authors have dealt with the subject of leadership of the care pathway (k = 9). The importance of defining a leader for each step of the care pathway was noted [25]. The lack of coordination without a responsible actor has been shown, especially when the care pathway includes actors in several contexts such as primary care [3]. Thus, new roles have been defined, such as case managers, joint program or nurse coordinators [4, 15, 42, 65], roles that enhance coordination among providers through the improvement of the continuity and quality of the information as well as communication [15].

More recently, the integration of services (k = 9) has been addressed. Because the care pathway approach can involve multiple partnerships between organizations and primary care, it is essential to integrate all stakeholders. The integration needs to be both organizational, at the macro and meso-level through shared purpose and priorities [4, 57, 25] and shared governance mechanisms [4, 12, 14, 59], and functional at the micro level through communication mechanisms and tools [4, 12, 14]. The unifying element is discussed between the shared interest for the patient [56, 57] or the outcomes [12] to align strategic goals. For Louis et al. [56], achieving shared purpose is part of the structural context.

Finally, the care pathway is seen as a means of health knowledge management (k = 7) that optimizes quality, efficiency, and organization [68, 70, 72]. But this topic, although strongly addressed between 2011 and 2012, did not seem to be unanimously agreed upon because it was not very well addressed afterwards. However, particular attention can be paid to the elicitation and integration of the contextual knowledge of the various actors involved throughout the care pathway into daily healthcare routine [3, 70, 72].

#### Attribute theme 5: The structural context of the system and organizations

Firstly, the local physical context (k = 10), topical in the recent literature, includes both the number of units and their positions [12, 67], but also the variety of services offered [13], and can be either an asset in terms of choice and accessibility or a constraint becoming a source of delay [14]. These barriers are important as the pathway crosses several formal healthcare organizations or informal care settings [24]. Therefore, the challenge of service integration has become essential [51].

Secondly, the availability of resources (k = 10) (human, material and financial) has a direct impact on the care pathway and the ability to meet the needs of the population [2, 62, 25]. A lack of adequate resources is an obvious obstacle to care pathways [50]. A lack of material and human resources, such as the availability of time at each service point [52, 53], or the lack of an electronic medical record [5], meant the unnecessary repetition of history taking, examinations and full investigations. From a financial point of view, the financial and personal resources that people have, are also key to determinants of the care pathways followed by patients [51].

Thirdly, the social context (k = 7) is less addressed in the current literature but has shown rapid growth in recent years. Social structure includes material and social resources including roles, rules, norms, and values [3, 24, 53, 68]. Some authors consider the social context as regularities of perception, behavior, belief and value that are expressed as customs, habits, patterns of behavior and other cultural artifacts [68]. Other authors consider that social structures shape people’s actions and that through people’s interactions they can then reproduce or change these social structures [53]. While others consider, for their part, that social and physical contexts can be at the origin of boundaries that mitigate against collaboration, adding to the complexity of shared clinical practices in this field [3, 24].

#### Attribute theme 6: The special role of the information system and data management

Data management (k = 14) plays an increasingly important role in the analysis and improvement of care pathways. The implementation of a care flow management system aligned to clinical workflows [67, 69], allows real-world data to be used [51], and visualized through performance dashboards to generate timely corrective action [4]. It also enables the analysis and monitoring of the variance in time and space within care pathways [43]. It is considered responsible for the rise of accountability [12, 75].

The Electronic Health Record system is a support tool (k = 13) in several aspects. Numerous authors consider that it supports the patient-centered approach [51, 67]. In particular, it has the capacity to support communication between health professionals, and between them and the patient [5, 12, 65, 67, 73, 75], but also to support healthcare knowledge learning [67, 73], and integrate clinical decision support into IT applications and clinical workflows [70]. This support throughout the care pathway can improve the quality of care and health outcomes by reducing medication errors and unnecessary investigations [5]. As stated by Fung-Kee-Fung et al. [4], the information system provides the fundamental connectivity across silos and professional groups to support the creation of care pathways and sustainable change at the system level.

The issue of digitalization (k = 5) has been treated very recently. It raises the issue of system integration throughout the care pathway. Despite the technological advances and the support of international organizations such as the guidelines on evidence-based digital health interventions for health system strengthening released by the WHO [76], there are still inefficiencies associated with trying to integrate EHRs across organizations [56]. These are frequently due to the use of different technological solutions by different stakeholders [30]. The challenge is therefore to propose a model for integrating information systems throughout the care pathway that are accessible to all stakeholders including patients themselves [4, 50, 51, 65].

#### Attribute theme 7: The advent of the learning system

Although it was not frequently addressed, some authors have developed, very recently, the importance of setting up a learning system (k = 3) to support the care pathway. Resulting from the work of Quinn [77] and Senge [78], it consists of the development of a system to learn from itself and its past experience and improve the effectiveness, efficiency, safety, and patient and family/caregiver experiences [65] through a feedback loop [24]. Data on outcomes can be used as feedback to identify improvement opportunities at various stages of the process or at specific interfaces between stakeholders. The learning system promotes “individual competence, systems thinking, cohesive vision, team learning, and integrating different perspectives” [4].

#### Related concepts

**Fig. 3:**
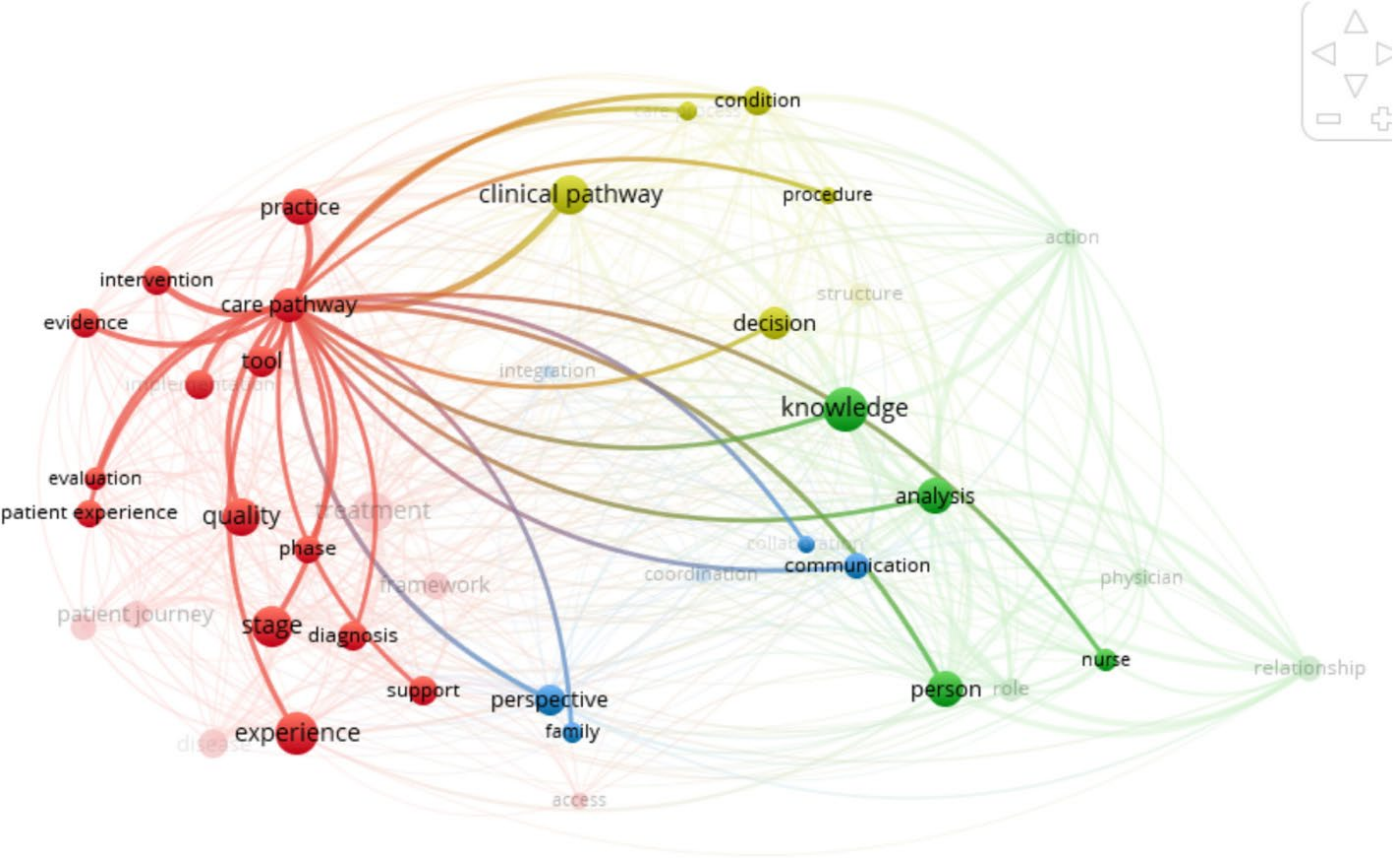
Care pathway bibliometric links

The related concepts are confusingly close or even integrated with the main concept studied [40]. Given the complexity of the use of concepts, we have relied, in addition to definitions found on an analysis of a bibliometric network by integrating all 44 articles, excluding abstracts and bibliographies, into the Vosviewer® software (version 2020). The results help us to refine our understanding of the concepts which define the links between the different keywords. The care pathway bibliometric links are provided as a comparator (see Fig. 3).

**Fig. 4:**
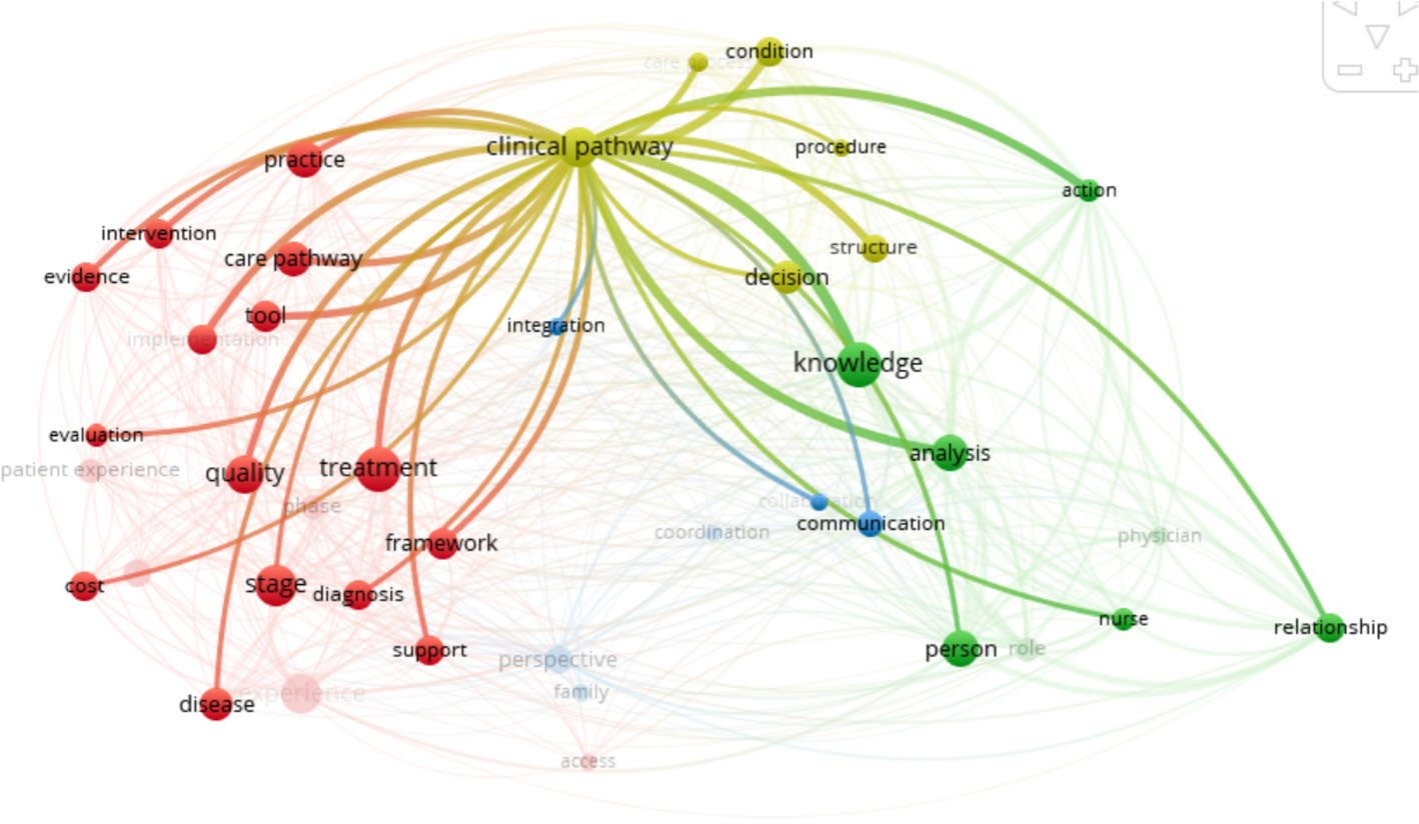
Clinical pathway bibliometric links

Clinical pathway (Fig. 4) was initially defined by De Bleser et al. [38]. It is a multidisciplinary intervention that aims to integrate the guidelines into daily routine and manage medical activities in order to improve the quality of service and optimize the use of resources [70]. It integrates a process of care approach [72] and aims at standardize care on a procedure or an episode of care [38, 49, 68], integrating decision-making supported by knowledge. What differentiates it from the care pathway is that it is restrained in time and is anchored in an organization [25], or even a service, and does not deal with the patient experience in any way. Clinical pathways are thus integrated in care pathways at the local level and focus on a single phase of care.

**Fig. 5:**
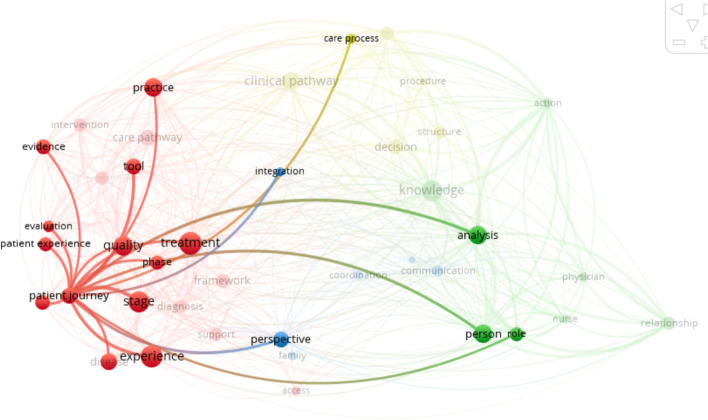
Patient journey bibliometric links

Patient journey (Fig. 5) consisted of sequential steps in the clinical process of the patient through their experience. It can be defined as “the spatiotemporal distribution of patients’ interactions with multiple care settings over time” [24]. By analyzing and mapping the patient experience from their perspective [5, 14, 57, 58, 71], the objective is to improve the quality of the service provided [14, 52]. In this approach, the patient journey is an integral part, and an essential component, of the care pathway. Although it also integrates the process approach, it is not linked to decision-making or knowledge management and does not consider structural constraints or the perception of the providers.

**Fig. 6:**
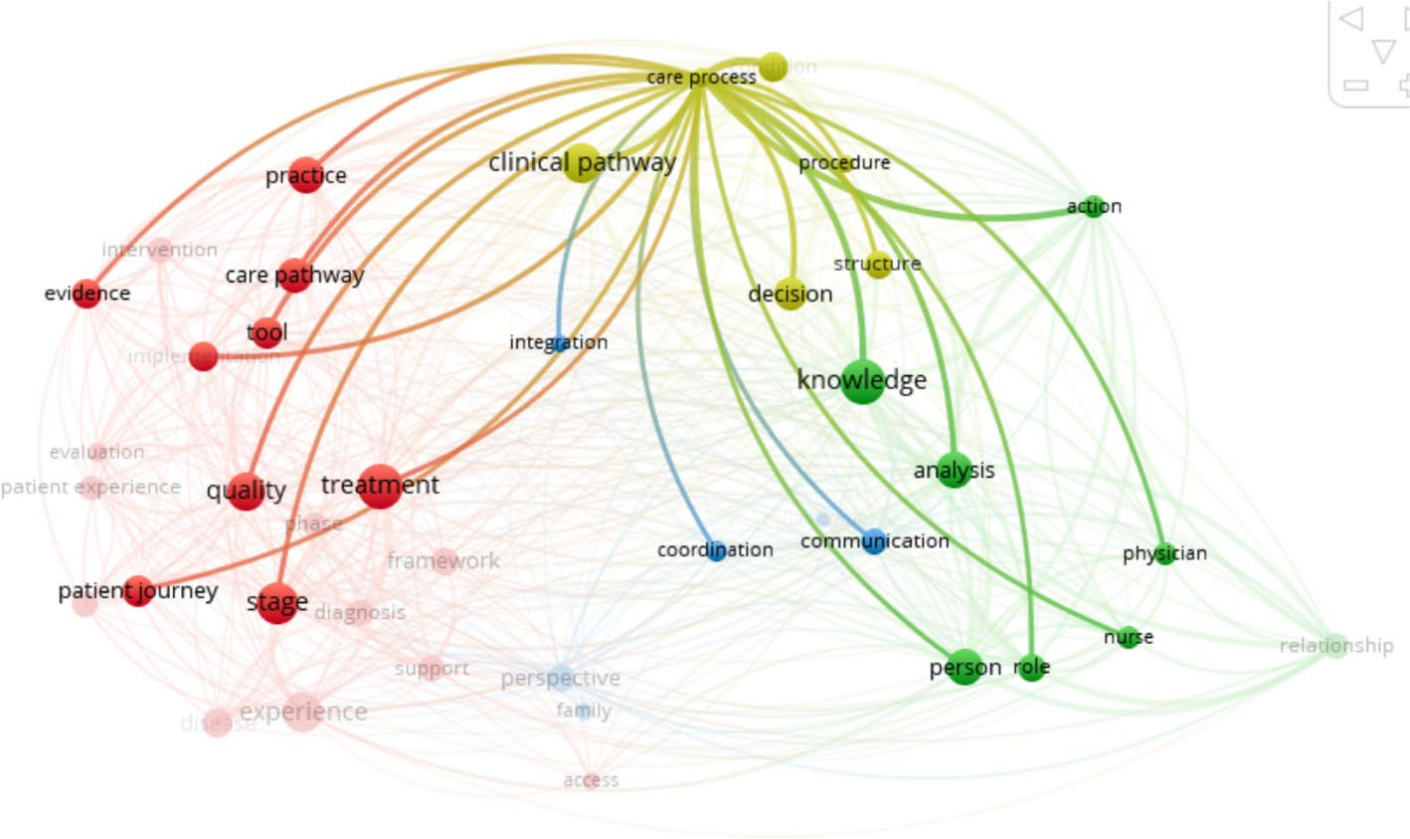
Care process bibliometric links

Finally, the care process (Fig. 6) is involved across the care continuum to standardize and streamline end-to-end care using management tools [4]. It is directly linked to the care pathway, the clinical pathway and the patient journey. However, although it supports coordination through decision-making and knowledge management, it does not consider the patient experience, the social relationships and the social dynamics. So, the care process is an integral part of the care pathway but does not consider all the characteristics of the latter.

#### Antecedents of the concept

Antecedents are events occurring or in place before the concept can emerge [40]. Our analysis has highlighted several prerequisites for care pathway implementation (see Additional file 4).

Firstly, several authors have stressed the importance of the availability of managerial skills (k = 10). They recommend the creation of a change management team [49, 55] consisting of a multidisciplinary team integrating not only knowledge about care pathways [60, 70], but also knowledge about operations research, information systems and industrial engineering [49, 55]. In addition, some authors advocate the presence of key change leaders in the group included clinicians, administrators, IT leaders, process experts, data analysts, nurses, and patient and family members [4, 24]. The project leaders must be available on a long-term basis [50, 75], have the ability to understand system interdependencies [24] and have the ability to create a safe learning environment in which openness is encouraged and everyone’s opinion is valued [3, 50]. This could be achieved by using consensus-driven approaches that could address institutional process barriers, resistance to change, and conflicting targets and priorities [4].

Secondly, care pathway projects should have a priori the adequate resources (k = 4), but their availability must be verified [62, 75]. The presence of an EHR is necessary to have access to reliable data at the pre-analysis phase and during the implementation phase to identify the relationships between the context, the mechanisms and the results obtained [2, 73].

Finally, other key success factors emerged from the literature (k = 10). Some authors noted that rules of co-involvement and a bottom-up strategy was needed [55]. Other authors emphasized that the selection of areas where there were clearly established deficiencies was essential given the cost of such projects, but also that the identification of any subgroups for whom its use may not be appropriate, was also required [73]. They highlighted the importance of following guidelines to achieve professional adherence [2, 50, 62, 72, 73], while maintaining flexibility in the approach to implementing a care pathway improvement program [62]. They also pointed to the importance of communicating on the progress of the project [50] and of monitoring the applicability of daily work tasks [73]. Finally, they consider it essential to embed the pathway into policy and strategy [2, 50, 72, 75]. While others, for their part, highlighted the importance of defining an iterative feedback loop for individuals and aggregated operational and clinical data [4, 24].

#### Consequences (outcomes) and identification of empirical referents

Consequences are events that are the results of the mobilization of the concept [40] and empirical referents, for their part, consist of observable phenomena by which defining attributes are recognized [40] (see Additional file 4). In a larger sense, this could be the Key Performance Indicators (KPIs) by which one can recognize the defining attributes and their outcomes.

Although the terms of quality and safety, efficiency and process improvement were the first themes in terms of aims, the most frequently occurring theme in the findings pertained to effects on the patient experience (k = 16). These were measured in different ways, including the impact of waiting times (k = 10), patient satisfaction (k = 7) and the patient quality of life (QALYs) (k = 4). There were also attempts to analyze the patient experience more broadly (k = 5), and to integrate patient needs into the redesign of the care pathway [5, 13, 56].

Efficiency of care (k = 15) was strongly supported by some authors as a desired outcome in care pathways. This outcome was first seen, as an objective, through the costs and cost effectiveness of programs [49, 55, 61, 70], however, more recently it has been considered a consequence of process improvements, rather than a program objective. It has been clearly defined as the reduction of costs through the reduction of the use of healthcare services [57]. Moreover, reduction in time spent in care, such as the length of stay or cycle time [2, 55], is commonly the consequence of process improvements.

Quality of care (k = 11) was addressed but much less frequently than expected. In the global approach, time to diagnostic is a good empirical referent to analyze the capacity of the first steps of the care pathway [4, 69]. Other referents such as reduction of unnecessary investigations and medication errors are also addressed but the number and types of complaints were addressed only by socio-political authors [53].

Health outcomes (k = 11) were also proposed but only since 2009 [73]. Clinical outcomes and mortality rates are empirical referents that are unanimously accepted. Recovery time and readmission rates were less frequently considered. Single disease index evaluation was proposed by very few authors [49, 70].

Process metrics and patient flow (k = 11) was addressed but only the execution time was unanimously accepted as an empirical referent. Apart from the process variance which is shared, only few authors have developed other KPIs such as the percentage of pathway completion [70], and evaluation for the reasons of pathway failure [70].

The variance of practices (k = 9) was not frequently addressed as an empirical referent; however, this is one of the objectives of the care pathway addressed in the literature. The introduction of guidelines [2] aims to decrease the variation within or between practices (k = 3).

Continuity of care (k = 6) was poorly addressed, even though we might assume that this is one of the primary objectives of the care pathway. This may be due to the difficulty of providing tangible results given the duration of such interventions.

Some authors noted an improvement in documentation and data collection (k = 5), measured by rate of documentation [54], the ability to better understand resource adequacy (k = 3) and a better comprehension of the links between decision outcomes and process performance (k = 2).

Not defined as an outcome, the Human Resources metrics are proposed by some authors and notably diagnostic quality and referral appropriateness, professional competences and staffing levels. Only Carayon et al. [24] proposed to integrate the quality of working life as an indicator, based on the principle that well-being at work has a direct impact on individual performance and on the results of the care pathway.

Moreover, not present in the empirical references, the measure of the team relationship and coordination (k = 4) has been proposed by some authors, however, the type of indicator has not been clearly explained.

### An integrative definition and conceptual framework of patient-centered care pathways

Given the results of our systematic review and concept analysis and our main objective of defining an integrative framework, we suggest the following definition:

“A patient-centered care pathway is a long-term and complex managerial intervention adopting a systemic approach, for a well-defined group of patients who journey across the entire continuum of care, from prevention and screening to recovery or palliative care. This intervention:

- prioritizes the centricity of patients and caregivers by analyzing the patient experience through their needs and expectations, taking into account the need for information, education, engagement and involvement and integrates the patient relationships as a fundamental need.
- supports the roles of professional actors involved in the care pathway by developing adherence to the patient-centered care approach; working on interdisciplinarity through the development of skills, both technical and above all relational; the clarification of roles and responsibilities; and by taking into account the experience of professionals both in understanding the organizational constraints and their well-being at work.
- integrates a process of care approach through the modeling and improvement of the care pathway by continuously integrating the latest knowledge and information to support clinical decision-making and by defining feedback loops to continuously improve clinical and non-clinical process supported by operation management contained within process improvement methodology approaches;
- embeds coordination structures through: the implementation of best practices and the translation of guidelines into daily practice; the support of informational continuity through the integration of services at the systemic level; the implementation of knowledge management along the care continuum; and the identification of leaders at each step of the care pathway;
- adapts to the contexts of both the physical and social structures by integrating the human, material, economic and financial resource constraints, as well as the social dynamics of power and trust relationships;
- is supported by information systems and data management, enabled by digitalization, which ensure the flow of information within the right context at the right time and place, and allows the continuous integration of the latest knowledge into the care flow and the management of accessible data in real time to monitor and evaluate variances in practices and outcomes;
- promotes the development of a learning health system to support the care pathway.

The aim and shared goal of a care pathway is to meet the needs and expectations of patients through continuous improvement of patient experience, patient outcomes, quality and safety while taking into account operational and social realities of the system.”

We know that this definition is important but feel that there is a great need for clarification of this concept and how these interventions can be successful given the costs involved. Furthermore, we consider that the proper sequencing of the care pathway should be defined according to the following eight phases: (1) Prevention and screening; (2) Signs and symptoms; (3) Early detection; (4) Diagnostic; (5) Referral systems; (6) Treatment; (7) Follow-ups; (8) Reeducation or Palliative care. In this way, the development of recognized KPIs enabling international comparisons of care pathways should finally make it possible to share knowledge and improve care pathways.

**Fig. 7:**
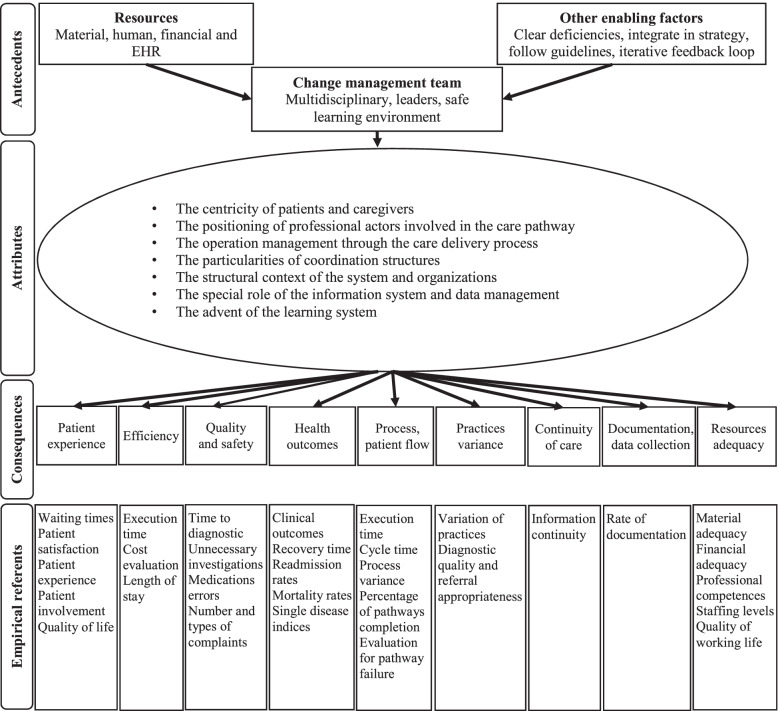
Integrative conceptual framework of care pathway

According to this definition and based on the literature review, we propose the following integrative conceptual framework illustrated in Fig. 7.

## Discussion

Using systematic review, concept analysis and bibliometric analysis, it was possible to develop a detailed understanding of the care pathway concept enabling us to propose an integrative conceptual framework and definition to try to meet the need for an international consensus and thus enabling international comparisons and improvement of care pathways.

The results of our work have highlighted the evolution and advances of the various uses of care pathways. Initially focused more on an organizational approach, there is growing support in the literature for a holistic approach that addresses the entire care across the continuum at the system level [4, 24, 42, 60]. Thus, patient centeredness has become the primary focus as more and more authors focus on the patient experience as the unit of quality analysis. In doing so, they have given greater importance to social relationships and especially to the relationship as a basic need and highlighted the need to design the service line structures mirroring patients’ needs [56]. They therefore approach the patient, not only as the individual who follows the pathway, but as a social being who has needs and expectations to fulfill, making meeting the needs and expectations of the patient and caregivers the core of the care pathway [24, 50, 51, 57]. However, the evaluation of the quality of healthcare services by the patient still raises several methodological questions to finally go beyond the simple consideration of satisfaction. Finally, patient and public involvement and patient engagement are also important issues to the point that some authors see a real power struggle between patients and clinicians [53] that can lead to tokenistic involvement.

The professional actors involved in the care pathway are naturally essential players, both because of their professional competencies and their ability to orient themselves towards the needs of the patient. However, they are also often part of a neglected factor. Some authors have shown one of the key criteria for the potential failure of care pathways is a failure to take into account the prevailing social dynamics and the importance of the buy-in of all stakeholders [65]. Moreover, some authors insist on the importance of the actors involved in the pathway to both integrate the social dynamics and confront the patient’s needs with operational realities and organizational constraints [24].

The operation management of process approach to care delivery also raises many challenges. Thus, some authors have developed tools for modeling and improving care processes by applying them in a systemic approach to incorporate clinical decision support into the modeling method [60]. This issue of continuous integration of updated guidelines into care pathways is indeed a major challenge given the rapid evolution of knowledge and the limited capacity of professionals to continuously integrate new knowledge. In addition, data simulation and data analysis methods coupled with process improvement methods are undeniable contributions to improve the issue of fluidity of processes and therefore the overall performance [49]. However, one of the pitfalls of staying focused on the process would be a failure to consider the social dimension, particularly the prevailing social dynamics.

Coordination structures are one of the points of improvement in the systemic approach. Ensuring the continuity of information along the care pathway, as well as having a formal leader for each portion of the pathway, would solve many of the problems of path breaks or unnecessary repetition of exams that cause unnecessary costs [5, 56, 59]. This begins with the implementation of a single information system and the integration of IT infrastructures across the entire care pathway at the system level and accessible to care professionals as well as patients and caregivers [4, 50, 51, 65].

The structural context of the system and organizations cannot be neglected because it directly impacts the results of the implementation of the care pathway. Firstly, because some physical constraints such as distances between several organizational entities [12, 14] can only be solved by major transformations in the infrastructures or in the initial process. Secondly, because failing to consider the dominant social dynamics could immediately call into question the entire care pathway intervention [3, 24] by implementing only cosmetic changes and not transforming clinical, administrative and organizational practices in a sustainable manner.

The information system plays a special role in care pathway, not only because it is the support of the informational continuity, but also because it enables real-time data analysis to support decision-making within the care pathway in the form of feedback loops [4, 24, 51].

Finally, it seems clear that care pathway programs at the systemic level are one potential intervention which could benefit from the implementation of a learning system [4]. Care pathway outcome data can be used as feedback to identify improvement opportunities at various stages of the process or at specific interfaces between stakeholders. This approach makes it possible to support the continuous improvement of the care process.

Given the richness of the contributions of the last 20 years, we advocate an integrated approach resulting in a fine-grained and comprehensive understanding of care pathway. Our proposal is compatible with the definition of Vanhaecht et al. [25] currently used by the EPA, but in our opinion, enriches it. It allows users to specify the operational realities to which stakeholders should pay attention. Moreover, it insists on adaptation to the social realities and the changes that inevitably accompany it and directly impact the success or failure. However, we were surprised that the approach to managing organizational change and transformation of practices were little addressed. Only Van Citters et al. [65] had noted that change management approaches were critical for successful care transformation and that they had been largely neglected in care pathways. We share this point of view and believe that care pathway intervention leaders must develop communicative action skills to support practices transformation. Not mentioned in the selected literature, we propose to enrich our conceptual framework of communicative action proposed by Habermas [79]. From our point of view, this dimension could explain the failures of such interventions or at least the difficulty in developing sustainable transformations in practices.

In general, the concept analysis approach has raised several questions about the depth of concept analysis and its place in knowledge advancement [80]. However, we believe that the combination of systematic review rigor and concept analysis richness, was necessary to meet the aims of this study and produced an integrated conceptual framework which is ready for use. However, this research has some limitations. Although interest is growing, few studies offer comprehensive empirical results on the deployment of a care pathway and its outcomes in a global systemic approach over the entire continuum of care. Moreover, there are a few examples of in-depth analysis of car pathways over a long period of time. Together, this means that the literature still offers little insight into potential outcomes of care pathways. Lastly, our analysis was limited to peer-reviewed articles; including other contributions such as theses and dissertations as well as grey literature could have brought out other categories or themes.

## Conclusion

This study has resulted in a fine-grained understanding of care pathways and in a clear definition relying on a powerful conceptual framework. It responds to a strong need for conceptual precision, as previous reviews have not addressed the care pathway on a systemic scale and in a holistic manner. In addition, our framework offers a holistic view of the pathway without being specific to a particular condition or context. Our framework encompasses 28 subcategories grouped into seven care pathway attributes that should be considered in complex care pathway intervention. It considers both operational and social realities and supporting the improvement and sustainable transformation of clinical, administrative, and organizational practices for the benefit of patients and caregivers, while taking into account professional experience, organizational constraints, and social dynamics. The formulation of these attributes, antecedents as success factors and consequences as potential outcomes, linked to their KPIs, allows the operationalization of this model for any pathway in any context. We believe that these results are of particular interest to policymakers, decision makers, managers and researchers alike, and that they could lead to an international consensus that would finally allow comparison of care pathway improvement programs. However, we consider that the development of a framework for analyzing the performance of such an intervention has yet to be developed in a more in-depth manner, such as by focusing on certain particularities of each phase so that managers and decision makers can rely on validated dashboards and KPIs. More empirical work needs to be done on the comprehensive approach, as defined in our proposed definition, to provide reliable results on the ability of these interventions to result in an overall improvement. In addition, the question of the understanding of social evaluation of the quality of care by the patient remains an open question, as the patient experience does not yet have conclusive KPIs as it is too often limited to patient satisfaction or QALYs.

## Supplementary Information

**Additional file 1.** Search strategy.

**Additional file 2.** PRISMA 2020 checklist.

**Additional file 3.** Quality appraisal of studies.

**Additional file 4.** Concept analysis coding.

## Data Availability

This systematic review is based on an analysis of 44 published papers which are all referenced within this manuscript. Data supporting our findings are included in the form of additional files.

## Abbreviations

EPA: European Pathway Association
IOM: Institute of Medicine of America
KPI: Key Performance Indicator
PRISMA: Preferred Reporting Items for Systematic reviews and Meta-Analyses
QALY: Quality Adjusted Life Year
WHO: World Health Organization
