# Supplementary material for: Definition and conceptualization of the patient-centered care pathway, a proposed integrative framework for consensus: a Concept analysis and systematic review
Source: BMC Health Serv Res. 2022 Apr 26;22:558. doi: 10.1186/s12913-022-07960-0 (PMC9040248; doi:10.1186/s12913-022-07960-0)
Supplement: Supplementary file 1 — Additional file 1. Search strategy. [file 12913_2022_7960_MOESM1_ESM.docx]

# Additional file 1

## Search strategy

**PubMed**

**Date of the search:** 21-04-2021

**Database limit:** No database limit has been apply

| **Concepts** | **#** | **Search strategy** | **Results** |
| --- | --- | --- | --- |
| Healthcare pathway (Controlled vocabulary) | 1 | "Patient Care Management"[Mesh:NoExp] OR "Workflow"[Mesh:NoExp] | 10,098 |
| Healthcare pathway (Free vocabulary) | 2 | Healthcare pathway*[TIAB] OR Clinical Processes[TIAB] OR medical processes[TIAB] OR healthcare processes[TIAB] OR Patient Journey[TIAB]  OR processes model*[TIAB] OR workflow management[TIAB] | 2,309 |
| Healthcare pathway (Combined) | 3 | #1 OR #2 | 12,270 |
| Healthcare access (Controlled vocabulary) | 4 | "Health Services Accessibility"[Mesh:NoExp] OR "Health Equity"[Mesh] OR "Waiting Lists"[Mesh:NoExp] OR "Time Factors"[Mesh:NoExp] | 1,280,419 |
| Healthcare access (Free vocabulary) | 5 | Health Services Access*[TIAB] OR Health Care Access*[TIAB] OR Health Services Availab*[TIAB] OR Access to Healthcare[TIAB] OR Health Equity[TIAB] OR Health inequit*[TIAB] OR Health disparities[TIAB] OR Wait* times[TIAB] OR Waiting Lists[TIAB] | 38,478 |
| Healthcare access (combined) | 6 | #4 OR #5 | 1,307,321 |
| Effectiveness (Controlled vocabulary) | 7 | "Patient Satisfaction"[Mesh:NoExp] OR "Patient Readmission"[Mesh] OR "Hospital Mortality"[Mesh] | 140,733 |
| Effectiveness (Free vocabulary) | 8 | Patient Satisfaction[TIAB] OR Patient needs[TIAB] OR Hospital Readmissions[TIAB] OR Patient Readmission[TIAB] OR 30 Day Readmission[TIAB] OR readmission rate[TIAB] OR Mortalit*[TIAB] OR Death rate[TIAB] | 854,571 |
| Effectiveness (combined) | 9 | #7 OR #8 | 936,302 |
| Efficiency as productivity (Controlled vocabulary) | 10 | "Operative Time"[Mesh] OR "Office Visits/statistics and numerical data"[Mesh:NoExp] OR "Referral and Consultation/statistics and numerical data"[Mesh:NoExp] OR "Bed Occupancy"[Mesh:NoExp] OR "Time-to-Treatment"[Mesh] OR "Time Management"[Mesh:NoExp] | 41,453 |
| Efficiency as productivity  (Free vocabulary) | 11 | Surgical Time*[TIAB] OR Operative Time*[TIAB] OR "Bed Occupancy"[TIAB]  OR Treatment Delay*[TIAB] OR Time-to-Treatment*[TIAB] OR Delayed Treatment*[TIAB] OR length of operation[TIAB] OR length of surgery[TIAB]  operat* time [TIAB] OR surgery time[TIAB] OR surgery duration[TIAB] OR patient referral[TIAB] OR therapy Delay*[TIAB] | 38,259 |
| Efficiency as productivity  (combined) | 12 | #10 OR #11 | 72,736 |
| Efficiency as economic (Controlled vocabulary) | 13 | "Costs and Cost Analysis"[Mesh:NoExp] OR "Cost-Benefit Analysis"[Mesh] OR "Healthcare Costs"[Mesh] OR "Direct Service Costs"[Mesh] OR "Cost Control"[Mesh] OR "Hospital Costs"[Mesh] | 201,783 |
| Efficiency as economic  (Free vocabulary) | 14 | Cost*[TIAB] | 640,920 |
| Efficiency as economic  (combined) | 15 | #13 OR #14 | 716,284 |
| Safety and quality  (Controlled vocabulary) | 16 | "Quality Assurance, Healthcare"[Mesh:NoExp] OR "Quality of Health Care"[Mesh:NoExp] OR "Total Quality Management"[Mesh:NoExp] OR "Quality Indicators, Health Care"[Mesh:NoExp] OR "Efficiency, Organizational"[Mesh] OR "Medical Errors"[Mesh:NoExp] OR "Medication Errors"[Mesh:NoExp] OR "Accidents, Occupational"[Mesh] | 210,821 |
| Safety and quality  (Free vocabulary) | 17 | Quality of Healthcare[TIAB] OR Healthcare Quality[TIAB] OR Continuous Quality Management[TIAB] OR Total Quality Management[TIAB] OR Six Sigma*[TIAB] OR hospital accidents[TIAB] OR medical error[TIAB] OR diagnostic error[TIAB] OR medication error[TIAB] OR surgical error[TIAB]  OR therapeutic error[TIAB] | 10,963 |
| Safety and quality  (combined) | 18 | #16 OR #17 | 216,480 |
| Characteristics of Staff  (Controlled vocabulary) | 19 | "Job Satisfaction"[Mesh:NoExp] OR Personnel Turnover[Mesh:NoExp] OR Personnel Loyalty[Mesh:NoExp] OR "Workload"[Mesh] OR Absenteeism[Mesh:NoExp] OR Presenteeism[Mesh:NoExp] OR "Shift Work Schedule"[Mesh] | 57,673 |
| Characteristics of Staff  (Free vocabulary) | 20 | Job satisfaction[TIAB] OR work satisfaction[TIAB] OR physician* satisfaction[TIAB] OR nurs* satisfaction[TIAB] OR allied health* satisfaction[TIAB] OR staff Loyalty[TIAB] OR staff turnover[TIAB] OR staff rentention[TIAB] OR Personnel Turnover[TIAB] OR Personnel rentention[TIAB] OR Personnel Loyalty[TIAB] OR employee rentention[TIAB] OR employee turnover[TIAB] OR employee Loyalty[TIAB] OR Absenteeism[TIAB] OR Presenteeism[TIAB] OR Night Shift[TIAB] OR Rotating Shift[TIAB] OR overtime[TIAB] OR workload[TIAB] | 80,766 |
| Characteristics of Staff  (combined) | 21 | #19 OR #20 | 120,061 |
| Combination of concepts | 22 | #3 AND (#6 OR #9 OR #12 OR #15 OR #18 OR #21) | 4,243 |

**Embase (Embase.com)**

**Date of the search:** 21-04-2021

**Database limit:** Embase results only

| **Concepts** | **#** | **Search strategy** | **Results** |
| --- | --- | --- | --- |
| Healthcare pathway (Controlled vocabulary) | 1 | 'healthcare management'/de OR 'managed care organization'/de | 31,359 |
| Healthcare pathway (Free vocabulary) | 2 | "workflow management":ti,ab,kw OR "Healthcare pathway$":ti,ab,kw OR "Patient Journey":ti,ab,kw OR ((Clinical OR medical OR Healthcare OR model*) NEAR/3 processes):ti,ab,kw | 14,930 |
| Healthcare pathway (Combined) | 3 | #1 OR #2 | 46,157 |
| Healthcare access (Controlled vocabulary) | 4 | 'healthcare access'/de OR 'health equity'/de OR 'time factor'/de OR 'hospital admission'/de | 307,606 |
| Healthcare access (Free vocabulary) | 5 | (Health NEAR/2 (Access* OR Availab* OR Equity OR inequit* OR disparities)):ti,ab,kw OR "Wait* times":ti,ab,kw OR "Waiting Lists":ti,ab,kw | 67,971 |
| Healthcare access (combined) | 6 | #4 OR #5 | 360,685 |
| Effectiveness (Controlled vocabulary) | 7 | 'patient satisfaction'/de OR 'hospital mortality'/de OR 'hospital readmission'/de | 239,961 |
| Effectiveness (Free vocabulary) | 8 | (Patient NEAR/2 (satisfaction OR needs)):ti,ab,kw OR Mortalit*:ti,ab,kw  OR "Death rate":ti,ab,kw OR ((Hospital OR Patient OR "30 Day" OR rate) NEAR/2 readmission$):ti,ab,kw | 1,268,791 |
| Effectiveness (combined) | 9 | #7 OR #8 | 1,395,359 |
| Efficiency as productivity (Controlled vocabulary) | 10 | 'operation duration'/de OR 'hospital bed utilization'/de OR 'time to treatment'/de OR 'time management'/de OR 'consultation'/de OR 'patient referral'/de OR 'therapy delay'/de | 374,406 |
| Efficiency as productivity  (Free vocabulary) | 11 | ((operat* OR surgery OR surgical) NEAR/2 (time$ OR duration OR length)):ti,ab,kw OR "Bed Occupancy":ti,ab,kw OR "Time-to-Treatment":ti,ab,kw OR "patient referral":ti,ab,kw OR ((Treatment$ OR therapy) NEAR/2 Delay*):ti,ab,kw | 211,150 |
| Efficiency as productivity  (combined) | 12 | #10 OR #11 | 497,452 |
| Efficiency as economic (Controlled vocabulary) | 13 | 'cost'/de OR 'healthcare cost'/de OR 'hospital cost'/exp OR 'cost control'/de OR 'cost benefit analysis'/de OR 'cost effectiveness analysis'/de | 509,663 |
| Efficiency as economic  (Free vocabulary) | 14 | Cost*:ti,ab,kw | 871,016 |
| Efficiency as economic  (combined) | 15 | #13 OR #14 | 1,090,146 |
| Safety and quality  (Controlled vocabulary) | 16 | 'healthcare quality'/de OR 'medical error'/exp OR 'total quality management'/de OR 'organization and management'/de OR 'productivity'/de OR 'occupational accident'/de | 868,721 |
| Safety and quality  (Free vocabulary) | 17 | ((medical OR diagnostic OR therapeutic OR medication OR surgical) NEAR/2 error):ti,ab,kw OR (Quality NEAR/2 Healthcare):ti,ab,kw OR ((Continuous OR total) NEAR/2 ("Quality Management")):ti,ab,kw OR "hospital accidents":ti,ab,kw OR "Six Sigma$":ti,ab,kw | 19,013 |
| Safety and quality  (combined) | 18 | #16 OR #17 | 875,097 |
| Characteristics of Staff  (Controlled vocabulary) | 19 | 'job satisfaction'/de OR 'personnel management'/de OR 'absenteeism'/de OR 'presenteeism'/de OR 'shift work'/exp OR 'working time'/de OR 'workload'/de | 152,737 |
| Characteristics of Staff  (Free vocabulary) | 20 | ((Job OR Work OR physician$ OR nurs* OR "allied health$") NEAR/2 satisfaction):ti,ab,kw OR ((staff OR Personnel OR employee) NEAR/2 (Loyalty OR turnover OR rentention)):ti,ab,kw OR ((Night OR Rotating) NEAR/2 Shift):ti,ab,kw OR Absenteeism:ti,ab,kw OR Presenteeism:ti,ab,kw OR overtime:ti,ab,kw OR workload:ti,ab,kw | 73,538 |
| Characteristics of Staff  (combined) | 21 | #19 OR #20 | 184,168 |
| Combination of concepts | 22 | #3 AND (#6 OR #9 OR #12 OR #15 OR #18 OR #21) | 20,144 |
| Embase results only | 23 | #22 AND [embase]/lim NOT ([embase]/lim AND [medline]/lim) | 7,111 |

**ABI/Inform**

**Date of the search:** 21-04-2021

**Database limit:** Limit results to academic publications

| **Concepts** | **#** | **Search strategy** | **Results** |
| --- | --- | --- | --- |
| Healthcare pathway (Controlled vocabulary) | 1 | SU("Patient care planning") | 465 |
| Healthcare pathway (Free vocabulary) | 2 | TI,AB("workflow management" OR "Healthcare pathway?" OR "Patient Journey") OR TI,AB((Clinical OR medical OR Healthcare OR model*) NEAR/3 processes) | 25 444 |
| Healthcare pathway (Combined) | 3 | 1 OR 2 | 25 897 |
| Healthcare access (Controlled vocabulary) | 4 | SU("healthcare access" OR "Medical waiting lists") | 4 743 |
| Healthcare access (Free vocabulary) | 5 | TI,AB((Health OR "Healthcare") NEAR/2 (Access* OR Availab*)) OR TI,AB(Health NEAR/2 (Equity OR inequit* OR disparities)) OR TI,AB("Wait* times" OR "Waiting Lists") | 14 194 |
| Healthcare access (combined) | 6 | 4 OR 5 | 17 590 |
| Effectiveness (Controlled vocabulary) | 7 | SU(Mortality OR "Patient Satisfaction") | 21 608 |
| Effectiveness (Free vocabulary) | 8 | TI,AB(Patient NEAR/2 (satisfaction OR needs)) OR TI,AB((Hospital OR Patient OR "30 Day" OR rate) NEAR/2 readmission$) OR TI,AB(Mortalit* OR "Death rate") | 33 009 |
| Effectiveness (combined) | 9 | 7 OR 8 | 43 291 |
| Efficiency as productivity (Controlled vocabulary) | 10 | SU("Time management" OR "Medical referrals") | 5 798 |
| Efficiency as productivity  (Free vocabulary) | 11 | TI,AB((operat* OR surgery OR surgical) NEAR/2 (time? OR duration OR length)) OR TI,AB((Treatment? OR therapy) NEAR/2 Delay*) OR TI,AB("Bed Occupancy" OR "Time-to-Treatment" OR "patient referral") | 8 704 |
| Efficiency as productivity  (combined) | 12 | 10 OR 11 | 14 465 |
| Efficiency as economic (Controlled vocabulary) | 13 | SU(Costs OR "Benefit cost analysis" OR "Cost analysis" OR "healthcare expenditures" OR "Hospital costs" OR "Cost control") | 489 564 |
| Efficiency as economic  (Free vocabulary) | 14 | TI,AB(Cost*) | 1 062 839 |
| Efficiency as economic  (combined) | 15 | 13 OR 14 | 1 308 330 |
| Safety and quality  (Controlled vocabulary) | 16 | SU("Quality control" OR "Quality of care" OR "Total quality" OR Efficiency OR "Medical errors" OR "Occupational accidents") | 162 388 |
| Safety and quality  (Free vocabulary) | 17 | TI,AB((medical OR diagnostic OR therapeutic OR medication OR surgical) NEAR/2 error) OR TI,AB(Quality NEAR/2 Healthcare) OR TI,AB((Continuous OR total) NEAR/2 ("Quality Management")) OR TI,AB("hospital accidents" OR "Six Sigma$") | 15 577 |
| Safety and quality  (combined) | 18 | 16 OR 17 | 168 035 |
| Characteristics of Staff  (Controlled vocabulary) | 19 | SU("Job satisfaction" OR "Employee turnover" OR Workloads OR Absenteeism OR "Shift work" OR Overtime) | 45 347 |
| Characteristics of Staff  (Free vocabulary) | 20 | TI,AB((Job OR Work OR physician? OR nurs* OR "allied health?") NEAR/2 satisfaction) OR TI,AB((staff OR Personnel OR employee) NEAR/2 (Loyalty OR turnover OR rentention)) OR TI,AB((Night OR Rotating) NEAR/2 Shift) OR TI,AB(Absenteeism OR Presenteeism OR overtime OR workload) | 48 335 |
| Characteristics of Staff  (combined) | 21 | 19 OR 20 | 72 938 |
| Combination of concepts | 22 | 3 AND (6 OR 9 OR 12 OR 15 OR 18 OR 21) | 5 633 |
| Limit results to academic publications | 23 | n/a | 3 927 |
