# Supplementary material for: Definition and conceptualization of the patient-centered care pathway, a proposed integrative framework for consensus: a Concept analysis and systematic review
Source: BMC Health Serv Res. 2022 Apr 26;22:558. doi: 10.1186/s12913-022-07960-0 (PMC9040248; doi:10.1186/s12913-022-07960-0)
Supplement: Supplementary file 3 — Additional file 3. Quality appraisal of studies. [file 12913_2022_7960_MOESM3_ESM.docx]

**Additional file 3**

## Quality appraisal of studies

| **Qualitative studies** | [1] | [5] | [13] | [14] | [15] | [30] | [40] | [41] | [48] | [51] | [54] | [56] | [57] | [58] | [62 | [64] | [65] | [66] | [67] | [69] | [70] | [72] | [73] |
| --- | --- | --- | --- | --- | --- | --- | --- | --- | --- | --- | --- | --- | --- | --- | --- | --- | --- | --- | --- | --- | --- | --- | --- |
| Philosophy congruent with methodology | U | U | + | + | U | U | U | U | U | + | + | U | U | - | U | U | U | + | U | U | U | U | U |
| Methodology congruent with aim | + | + | + | + | + | + | + | - | + | + | + | + | + | + | + | + | + | + | + | + | + | - | + |
| Methodology congruent with data collection methods | + | + | + | + | + | + | + | + | + | + | + | + | + | + | + | + | U | U | + | + | + | - | U |
| Methodology congruent with analysis | + | + | + | + | + | + | + | + | + | + | + | + | + | + | + | + | U | U | + | + | U | - | U |
| Methodology congruent with interpretation of results | + | + | + | + | + | + | + | - | + | + | + | + | + | + | + | + | U | U | + | + | U | - | U |
| Researcher background state | - | - | - | + | - | - | - | - | - | + | + | - | - | - | - | - | - | - | - | - | - | - | - |
| Influence of researcher stated | - | - | - | + | - | - | - | - | - | - | + | - | - | - | - | - | - | - | - | - | - | - | - |
| Participant voices adequately represented | + | + | + | + | + | + | + | U | + | + | + | - | + | - | + | + | U | U | U | + | U | - | U |
| Ethical approval | + | + | - | + | + | - | + | + | + | + | + | - | + | - | + | + | - | - | - | + | - | - | - |
| Conclusions derived from data | + | + | + | + | + | + | + | + | + | + | + | + | + | + | + | + | + | U | + | U | U | - | U |
| **Survey studies** | [50] | [52] | [60] |  |  |  |  |  |  |  |  |  |  |  |  |  |  |  |  |  |  |  |  |
| Clear aim addressed | + | + | + |  |  |  |  |  |  |  |  |  |  |  |  |  |  |  |  |  |  |  |  |
| Design appropriate for research question | + | + | + |  |  |  |  |  |  |  |  |  |  |  |  |  |  |  |  |  |  |  |  |
| Clear selection process | + | - | + |  |  |  |  |  |  |  |  |  |  |  |  |  |  |  |  |  |  |  |  |
| Potential for selection bias | + | - | - |  |  |  |  |  |  |  |  |  |  |  |  |  |  |  |  |  |  |  |  |
| Subjects are representative | + | - | + |  |  |  |  |  |  |  |  |  |  |  |  |  |  |  |  |  |  |  |  |
| Power analysis included | - | - | + |  |  |  |  |  |  |  |  |  |  |  |  |  |  |  |  |  |  |  |  |
| Response rate in % (satisfactory if ≥ 60%)* | U | - | + |  |  |  |  |  |  |  |  |  |  |  |  |  |  |  |  |  |  |  |  |
| Valid and reliable measurements | + | - | + |  |  |  |  |  |  |  |  |  |  |  |  |  |  |  |  |  |  |  |  |
| Statistical significance assessed | + | - | + |  |  |  |  |  |  |  |  |  |  |  |  |  |  |  |  |  |  |  |  |
| Confidence intervals given | - | - | - |  |  |  |  |  |  |  |  |  |  |  |  |  |  |  |  |  |  |  |  |
| Unaccounted confounding factors present | - | - | - |  |  |  |  |  |  |  |  |  |  |  |  |  |  |  |  |  |  |  |  |
| **Descriptive cross-sectional stud** | [34] | [53] | [59] | [61] |  |  |  |  |  |  |  |  |  |  |  |  |  |  |  |  |  |  |  |
| Clear aim addressed | + | + | + | + |  |  |  |  |  |  |  |  |  |  |  |  |  |  |  |  |  |  |  |
| Methods appropriate for research question | + | + | + | + |  |  |  |  |  |  |  |  |  |  |  |  |  |  |  |  |  |  |  |
| Sample representative | + | + | + | + |  |  |  |  |  |  |  |  |  |  |  |  |  |  |  |  |  |  |  |
| Measurements accurate and valid | + | + | + | + |  |  |  |  |  |  |  |  |  |  |  |  |  |  |  |  |  |  |  |
| Data collection method appropriate | + | + | + | + |  |  |  |  |  |  |  |  |  |  |  |  |  |  |  |  |  |  |  |
| Participant number large enough | + | + | + | + |  |  |  |  |  |  |  |  |  |  |  |  |  |  |  |  |  |  |  |
| Results correct presented | + | + | + | - |  |  |  |  |  |  |  |  |  |  |  |  |  |  |  |  |  |  |  |
| Analysis correct | + | + | + | U |  |  |  |  |  |  |  |  |  |  |  |  |  |  |  |  |  |  |  |
| Findings clearly stated | + | + | + | + |  |  |  |  |  |  |  |  |  |  |  |  |  |  |  |  |  |  |  |
| **Mixed-methods studies** | [3] | [4] | [55] | [63] |  |  |  |  |  |  |  |  |  |  |  |  |  |  |  |  |  |  |  |
| Qualitative objective present | + | + | + | + |  |  |  |  |  |  |  |  |  |  |  |  |  |  |  |  |  |  |  |
| Design/methods appropriate for research question | + | + | + | + |  |  |  |  |  |  |  |  |  |  |  |  |  |  |  |  |  |  |  |
| Context described | + | + | + | + |  |  |  |  |  |  |  |  |  |  |  |  |  |  |  |  |  |  |  |
| Participants described & sample justified | + | - | + | + |  |  |  |  |  |  |  |  |  |  |  |  |  |  |  |  |  |  |  |
| Qual data collection & analysis described | + | - | - | + |  |  |  |  |  |  |  |  |  |  |  |  |  |  |  |  |  |  |  |
| Researcher’s reflexivity described | - | - | - | - |  |  |  |  |  |  |  |  |  |  |  |  |  |  |  |  |  |  |  |
| Quant sampling & sample appropriate | U | U | - | + |  |  |  |  |  |  |  |  |  |  |  |  |  |  |  |  |  |  |  |
| Justification of measurements (validity) | - | - | - | + |  |  |  |  |  |  |  |  |  |  |  |  |  |  |  |  |  |  |  |
| Confounding variables controlled | - | - | - | - |  |  |  |  |  |  |  |  |  |  |  |  |  |  |  |  |  |  |  |
| Mixed-methods design justified by authors | + | + | + | + |  |  |  |  |  |  |  |  |  |  |  |  |  |  |  |  |  |  |  |
| Combination of qual & quant data collection & analysis | + | - | - | - |  |  |  |  |  |  |  |  |  |  |  |  |  |  |  |  |  |  |  |
| Integration qual & quant results | + | - | - | + |  |  |  |  |  |  |  |  |  |  |  |  |  |  |  |  |  |  |  |
| + = yes; - = no; U = unclear; NA = Criterion not applicable because of descriptive design; * 60% was chosen based on recommendations in the literature | | | | | | | | | | | | | | | | | | | | | | | |
