# Supplementary material for: Definition and conceptualization of the patient-centered care pathway, a proposed integrative framework for consensus: a Concept analysis and systematic review
Source: BMC Health Serv Res. 2022 Apr 26;22:558. doi: 10.1186/s12913-022-07960-0 (PMC9040248; doi:10.1186/s12913-022-07960-0)
Supplement: Supplementary file 4 — Additional file 4. Concept analysis coding. [file 12913_2022_7960_MOESM4_ESM.docx]

# Additional file 4

## Concept analysis coding

### Uses of care pathway

| **Subcategory** | **k** | **Concerned articles** |
| --- | --- | --- |
| Improve quality of care and safety | k=26 | [2] ; [3] ; [14] ; [24] ; [25] ; [30] ; [36] ; [47] ; [50] ; [52] ; [53] ; [55] ; [56] ; [57] ; [58] ; [60] ; [61] ; [63] ; [64] ; [66] ; [67] ; [68] ; [70] ; [71] ; [72] ; [73] |
| Improve efficiency of care delivery | k=24 | [2] ; [3] ; [4] ; [12] ; [13] ; [14] ; [25] ; [30] ; [36] ; [40] ; [48] ; [50] ; [52] ; [56] ; [57] ; [58] ; [59] ; [63] ; [67] ; [68] ; [70] ; [71] ; [72] ; [73] |
| Optimize the delivery process through a supply chain point of view | k=22 | [2] ; [13] ; [14] ; [24] ; [41] ; [47] ; [50] ; [52] ; [53] ; [55] ; [56] ; [57] ; [58] ; [61] ; [66] ; [67] ; [68] ; [69] ; [71] ; [72] ; [36] ; [73] |
| Integrate best practices through guidelines and evidence-based medicine | k=17 | [2] ; [4] ; [5] ; [36] ; [47] ; [53] ; [57] ; [58] ; [59] ; [60] ; [61] ; [63] ; [65] ; [67] ; [68] ; [71] ; [72] |
| Improve patient experience through patient journey analysis | k=17 | [2] ; [3] ; [4] ; [5] ; [12] ; [13] ; [14] ; [15] ; [24] ; [30] ; [34] ; [40] ; [51] ; [56] ; [64] ; [69] ; [71] |
| Developed the patient-centered care | k=13 | [1] ; [13] ; [25] ; [34] ; [40] ; [50] ; [53] ; [54] ; [62] ; [63] ; [65] ; [66] ; [72] |
| Improve patient outcomes | k=13 | [2] ; [5] ; [12] ; [13] ; [24] ; [48] ; [51] ; [56] ; [58] ; [68] ; [70] ; [71] ; [72] |
| Improving service delivery coordination | k=13 | [1] ; [2] ; [12] ; [24] ; [50] ; [54] ; [57] ; [58] ; [63] ; [64] ; [68] ; [71] ; [72] |
| Standardize care delivery | k=12 | [4] ; [13] ; [15] ; [36] ; [47] ; [52] ; [57] ; [58] ; [59] ; [60] ; [68] ; [72] |
| Reduce practice variation | k=9 | [2] ; [36] ; [47] ; [50] ; [53] ; [57] ; [68] ; [71] ; [73] |
| Improve and continuously assess performance | k=8 | [2] ; [30] ; [47] ; [53] ; [57] ; [58] ; [64] ; [71] |
| Meet the needs of patient | k=6 | [3] ; [15] ; [24] ; [33] ; [56] ; [69] |
| Improve interprofessional collaboration | k=5 | [48] ; [60] ; [66] ; [71] ; [73] |
| Support change | k=5 | [4] ; [33] ; [57] ; [67] ; [71] |
| Support clinical decision making | k=4 | [24] ; [47] ; [58] ; [67] |
| Improve communication | k=3 | [2] ; [63] ; [71] |
| Consider needs of health care workers | k=2 | [24] ; [73] |
| Improve referral system | k=2 | [5] ; [34] |
| Define shared purposes and meaningful objectives | k=2 | [2] ; [54] |
| Monitor staff compliance | k=1 | [71] |
| Support the knowledge management | k=1 | [70] |
| Improve patient and family member access to information | k=1 | [64] |
| Adopt a system approach | k=1 | [4] |
| Understand power dynamics and relational factors | k=1 | [51] |

### Attribute theme 1: Centricity of patients and their caregivers

| **Subcategory** | **k** | **Concerned articles** |
| --- | --- | --- |
| Patient experience | k=15 | [3] ; [5] ; [12] ; [13] ; [14] ; [15] ; [30] ; [40] ; [49] ; [50] ; [51] ; [56] ; [62] ; [63] ; [73] |
| Patient information and education | k=15 | [3] ; [5] ; [13] ; [14] ; [15] ; [34] ; [40] ; [49] ; [51] ; [55] ; [56] ; [62] ; [63] ; [69] ; [73] |
| Patient engagement | k=15 | [3] ; [14] ; [24] ; [25] ; [33] ; [48] ; [49] ; [51] ; [53] ; [54] ; [55] ; [56] ; [62] ; [63] ; [65] |
| Relationship as the basic need | k=9 | [3] ; [5] ; [13] ; [14] ; [15] ; [40] ; [51] ; [56] ; [62] |
| Patient and Public Involvement | k=9 | [12] ; [13] ; [33] ; [49] ; [51] ; [54] ; [56] ; [60] ; [62] |
| Individualized care | k=8 | [14] ; [33] ; [49] ; [53] ; [62] ; [63] ; [69] ; [72] |
| Psychosocial support | k=8 | [2] ; [3] ; [5] ; [14] ; [15] ; [40] ; [56] ; [60] |
| Include family and caregiver | k=8 | [14] ; [15] ; [48] ; [49] ; [55] ; [56] ; [62] ; [63] |

### Attribute theme 2: Positioning of professional actors involved in the care pathway

| **Subcategory** | **k** | **Concerned articles** |
| --- | --- | --- |
| Patient-centered care | k=18 | [1] ; [3] ; [13] ; [24] ; [25] ; [34] ; [40] ; [49] ; [50] ; [51] ; [53] ; [54] ; [62] ; [63] ; [65] ; [66] ; [72] ; [73] |
| Multidisciplinary team-working | k=17 | [2] ; [3] ; [24] ; [25] ; [36] ; [48] ; [50] ; [51] ; [54] ; [56] ; [57] ; [60] ; [66] ; [70] ; [71] ; [72] ; [73] |
| Staff skills | k=10 | [3] ; [5] ; [12] ; [13] ; [15] ; [48] ; [49] ; [51] ; [54] ; [62] |
| Enable professionals to examine their roles and responsibilities | k=6 | [2] ; [12] ; [25] ; [60] ; [63] ; [71] |
| Staff experience | k=2 | [24] ; [51] |

### Attribute theme 3: Operation management through the care delivery process

| **Subcategory** | **k** | **Concerned articles** |
| --- | --- | --- |
| Process approach of the care delivery | k=23 | [3] ; [4] ; [13] ; [14] ; [24] ; [25] ; [30] ; [36] ; [41] ; [47] ; [49] ; [50] ; [52] ; [54] ; [58] ; [59] ; [61] ; [65] ; [66] ; [67] ; [68] ; [71] ; [72] |
| Modeling and improving process methods | k=18 | [1] ; [3] ; [4] ; [5] ; [15] ; [30] ; [40] ; [41] ; [49] ; [52] ; [57] ; [58] ; [59] ; [63] ; [65] ; [66] ; [67] ; [69] |
| Process improvement methodology | k=11 | [4] ; [13] ; [25] ; [41] ; [47] ; [51] ; [58] ; [59] ; [61] ; [63] ; [72] |

### Attribute theme 4: Particularities of coordination structures

| **Subcategory** | **k** | **Concerned articles** |
| --- | --- | --- |
| Guidelines and evidence-based practices | k=24 | [2] ; [3] ; [5] ; [13] ; [25] ; [30] ; [33] ; [34] ; [36] ; [48] ; [49] ; [51] ; [53] ; [56] ; [57] ; [58] ; [64] ; [65] ; [66] ; [68] ; [70] ; [71] ; [72] ; [73] |
| Information continuity | k=13 | [1] ; [2] ; [5] ; [12] ; [36] ; [49] ; [54] ; [55] ; [57] ; [58] ; [60] ; [63] ; [73] |
| Leadership of the pathway | k=9 | [3] ; [4] ; [15] ; [25] ; [40] ; [54] ; [56] ; [60] ; [63] |
| Integration of services | k=9 | [4] ; [12] ; [14] ; [24] ; [25] ; [47] ; [54] ; [55] ; [57] |
| Knowledge management | k=7 | [3] ; [4] ; [63] ; [65] ; [66] ; [68] ; [70] |

### Attribute theme 5: Structural context of the system and organizations

| **Subcategory** | **k** | **Concerned articles** |
| --- | --- | --- |
| Physical structures | k=10 | [3] ; [5] ; [12] ; [13] ; [14] ; [24] ; [49] ; [50] ; [54] ; [65] |
| Resources (human, materials, financials) | k=10 | [2] ; [12] ; [25] ; [48] ; [49] ; [50] ; [51] ; [60] ; [63] ; [64] |
| Social context | k=7 | [2] ; [3] ; [24] ; [49] ; [51] ; [66] ; [71] |

### Attribute theme 6: The special role of the information system and data management

| **Subcategory** | **k** | **Concerned articles** |
| --- | --- | --- |
| Data management | k=14 | [4] ; [5] ; [12] ; [36] ; [41] ; [48] ; [49] ; [54] ; [56] ; [57] ; [65] ; [68] ; [71] ; [73] |
| Support tool | k=13 | [4] ; [5] ; [12] ; [48] ; [49] ; [54] ; [56] ; [57] ; [63] ; [65] ; [68] ; [71] ; [73] |
| Digitalization | k=5 | [4] ; [30] ; [48] ; [49] ; [56] |

### Attribute theme 7: The advent of the learning system

| **Subcategory** | **k** | **Concerned articles** |
| --- | --- | --- |
| Learning system | k=3 | [4] ; [24] ; [63] |

### Antecedents of the concept

| **Subcategory** | **k** | **Concerned articles** |
| --- | --- | --- |
| Managerial skills resources | k=10 | [3] ; [4] ; [24] ; [47] ; [51] ; [53] ; [58] ; [60] ; [68] ; [73] |
| Material resources | k=4 | [60] ; [64] ; [68] ; [73] |
| Other enabling factors | k=10 | [2] ; [4] ; [48] ; [53] ; [58] ; [60] ; [68] ; [70] ; [71] ; [73] |

### Consequences (outcomes)

| **Subcategory** | **k** | **Concerned articles** |
| --- | --- | --- |
| Effects on patient experience | k=16 | [1] ; [2] ; [3] ; [4] ; [5] ; [13] ; [14] ; [24] ; [36] ; [50] ; [51] ; [54] ; [56] ; [64] ; [69] ; [71] |
| Efficiency of care | k=15 | [2] ; [4] ; [12] ; [25] ; [36] ; [41] ; [47] ; [53] ; [56] ; [59] ; [62] ; [67] ; [68] ; [71] ; [72] |
| Quality of care | k=11 | [2] ; [24] ; [25] ; [36] ; [56] ; [59] ; [62] ; [66] ; [67] ; [68] ; [72] |
| Health outcomes | k=11 | [2] ; [41] ; [47] ; [50] ; [53] ; [55] ; [56] ; [57] ; [62] ; [64] ; [66] |
| Variance of practices | k=11 | [2] ; [4] ; [25] ; [36] ; [55] ; [62] ; [64] ; [66] ; [68] ; [71] ; [72] |
| Continuity of care and patient flow | k=9 | [1] ; [2] ; [3] ; [4] ; [52] ; [53] ; [57] ; [67] ; [68] |
| Documentation and data collection | k=5 | [2] ; [36] ; [47] ; [53] ; [57] |
| Resources adequacy | k=3 | [47] ; [50] ; [52] |
| Understanding the link between decision outcomes and process performance | k=3 | [58] ; [59] |

### Identification of empirical referents

|  | **Subcategory** | **k** | **Concerned articles** |
| --- | --- | --- | --- |
| Efficiency of care | Costs |  | [24] ; [25] ; [47] ; [50] ; [53] ; [56] ; [58] ; [59] ; [68] |
|  | Length of stay |  | [25] ; [41] ; [47] ; [51] ; [53] ; [56] ; [59] ; [63] ; [68] ; [71] ; [72] |
| Process metrics | Execution times |  | [4] ; [5] ; [13] ; [47] ; [50] ; [51] ; [53] ; [58] ; [72] |
|  | Percentage of pathways completion |  | [68] |
|  | Evaluation of the reasons for pathway failure |  | [68] |
|  | Process variance |  | [25] ; [47] ; [68] ; [72] |
|  | Rate of documentation |  | [52] |
| Quality metrics | Time to diagnostic |  | [4] ; [67] |
|  | Unnecessary investigations |  | [5] ; [24] |
|  | Medication errors |  | [5] ; [24] ; [71] |
|  | Number and types of complaints |  | [51] |
| Health outcomes | Single disease indices evaluation |  | [47] ; [68] |
|  | Clinical outcomes |  | [12] ; [25] ; [50] ; [51] ; [53] ; [56] ; [63] ; [64] |
|  | Recovery time |  | [41] ; [56] ; [71] |
|  | Readmission rate |  | [41] ; [53] ; [63] ; [71] |
|  | Mortality rate |  | [47] ; [51] ; [53] ; [63] ; [71] |
| HR metrics | Diagnostic quality and referral appropriateness |  | [58] ; [34] |
|  | Professional competences |  | [25] ; [52] ; [58] |
|  | Staffing levels |  | [47] |
|  | Quality of working life |  | [24] |
| Patient experience | Waiting times |  | [4] ; [13] ; [25] ; [41] ; [47] ; [50] ; [51] ; [53] ; [58] ; [71] |
|  | Patient satisfaction |  | [24] ; [25] ; [50] ; [56] ; [58] ; [64] ; [71] |
|  | Patient experience |  | [13] ; [14] ; [24] ; [50] ; [51] |
|  | Patients’ involvement |  | [25] ; [58] ; [63] |
|  | Quality of life |  | [47] ; [50] ; [56] ; [59] |
| Team indicators | Team relationship and coordination |  | [5] ; [25] ; [63] ; [71] |
